# CRA toolbox: software package for conditional robustness analysis of cancer systems biology models in MATLAB

**DOI:** 10.1186/s12859-019-2933-z

**Published:** 2019-07-09

**Authors:** Fortunato Bianconi, Chiara Antonini, Lorenzo Tomassoni, Paolo Valigi

**Affiliations:** 1 Independent Researcher, Belvedere 44, Montefalco, Perugia, 06036 Italy; 2 Department of Engineering, University of Perugia, G. Duranti 95, Perugia, 06132 Italy

**Keywords:** Ordinary differential equation models, Conditional robustness analysis, MATLAB package, Signaling networks

## Abstract

**Background:**

In cancer research, robustness of a complex biochemical network is one of the most relevant properties to investigate for the development of novel targeted therapies. In cancer systems biology, biological networks are typically modeled through Ordinary Differential Equation (ODE) models. Hence, robustness analysis consists in quantifying how much the temporal behavior of a specific node is influenced by the perturbation of model parameters. The Conditional Robustness Algorithm (CRA) is a valuable methodology to perform robustness analysis on a selected output variable, representative of the proliferation activity of cancer disease.

**Results:**

Here we introduce our new freely downloadable software, the CRA Toolbox. The CRA Toolbox is an Object-Oriented MATLAB package which implements the features of CRA for ODE models. It offers the users the ability to import a mathematical model in Systems Biology Markup Language (SBML), to perturb the model parameter space and to choose the reference node for the robustness analysis. The CRA Toolbox allows the users to visualize and save all the generated results through a user-friendly Graphical User Interface (GUI). The CRA Toolbox has a modular and flexible architecture since it is designed according to some engineering design patterns. This tool has been successfully applied in three nonlinear ODE models: the Prostate-specific *Pten*^−/−^ mouse model, the Pulse Generator Network and the EGFR-IGF1R pathway.

**Conclusions:**

The CRA Toolbox for MATLAB is an open-source tool implementing the CRA to perform conditional robustness analysis. With its unique set of functions, the CRA Toolbox is a remarkable software for the topological study of biological networks. The source and example code and the corresponding documentation are freely available at the web site: http://gitlab.ict4life.com/SysBiOThe/CRA-Matlab.

**Electronic supplementary material:**

The online version of this article (10.1186/s12859-019-2933-z) contains supplementary material, which is available to authorized users.

## Background

In Systems Biology, mathematical modeling and computational software are important tools to unravel the complexity of biological systems and predict their behavior under different perturbations [1]. Typically, many models consist of a set of Ordinary Differential Equations (ODEs) which allow understanding and reproducing the dynamic behavior of molecular interactions through simulations and integration of the ODEs [2]. To support mathematical modeling of biological networks, the use of software tools has grown substantially in recent years. These software are designed to assist the users at different stages of the modeling process, from model generation to parameter estimation and model analysis.

In cancer research, the use of systems biology approaches is particularly useful to elucidate mechanisms of tumorigenesis and tumor resistance. Computational predictive models, integrated with patient data, help scientists in the validation of new and durable therapies [3]. In order to discover effective and targeted drugs, robustness is one of the most relevant properties of cancer signaling networks to investigate. Robustness is defined as the ability of a biological system to maintain its functionalities against internal and external perturbations [4]. In more detail, cancer robustness is a quantitative measure of the tumor proliferation attitude against extracellular inputs. Thus, understanding new ways to reduce robustness of the cell proliferation activity is a key issue in cancer systems biology. Since cell growth is driven by protein interaction networks, the proliferation activity can be quantified by looking at the activation of a protein involved in the proliferation process [5]. In mathematical modeling, this can be done by perturbing model parameters and analyze how the concentration of the protein of interest changes over time. An algorithm developed for this purpose is the Conditional Robustness Algorithm (CRA) proposed in [5]. This algorithm, through computational perturbations and simulations, identifies a small number of nodes in the cancer network which influences most the activity of the proliferation indicator. As a result, by conditioning these nodes with specific drugs, it may be possible to reduce the tumor robustness.

Robustness of mathematical model problem is well studied in literature except when the models are based on nonlinear ODE. A class of methods is aimed at analyzing the geometry and the volume of the feasible region, which is the region in the parameter space that allows the system to properly work [6]. Moreover, there are other algorithms that infer the robustness of a model looking at its topology, such as the number and the structure of positive and negative loops [7]. However, these techniques are typically applied to mathematical models whose parameters are not kinetic. Another and different category of methods is the Global Sensitivity Analysis (GSA) class of algorithms [8]. They are similar to CRA and are involved in the analysis of the uncertainties in kinetic model parameters through the sampling of the parameter space. Despite that, GSA and CRA have clear distinct goals. GSA is typically interested in the variation of a performance index with the respect to the model parameters. Since many times this is basically implemented through the derivatives, GSA tools are useful when an optimization of the system is required [9]. In the CRA algorithm, on the other hand, the purpose is not to maximize or minimize a certain optimization function, but the main interest is in finding the conditioning set, which is the subset of parameters that more likely is able to impose a specific behavior to one output of the model.

In order to facilitate the study of cancer robustness and the application of CRA, we develop the CRA Toolbox, a software package for MATLAB. It is an open source tool which allows non expert users to apply the CRA to any ODE model in a simple and quick way. The CRA Toolbox consists in a easy to use Graphical User Interface (GUI) and in a set of functions which can be easily extended by the users in order to achieve specific requirements.

In the following subsection, we briefly describe the CRA method implemented in the toolbox. A detailed description of the method can be found in [5].

### Conditional Robustness Algorithm (CRA)

The main underlying theoretical principle of CRA is the definition of conditional robustness proposed by Kitano in [10]: it is the quantitative measure of the ability of a system S to maintain a specific property *τ* against some perturbations of the parameter vector *p* of S. The mathematical formulation is the following:

$$\mathbf{R}^{n}_{\tau,\mathbf{P}} := \int_{\mathbf{P}} f_{P}(p)\zeta_{\tau}^{S}(p) dp $$

where *f*_*P*_(*p*) is the probability density function of *p*, **P** is the parameter space and $\zeta _{\tau }^{S}(p)$ is a function that quantifies and represents the property *τ* that is under investigation.

The CRA is a stochastic approach for performing conditional robustness analysis of mathematical models, such as ODE models representing biochemical interaction networks. Its purpose is to quantify the influence of each model parameter on the behavior of a specific output node. Let denote with S the following ODE system:

$$S = \left\{ \begin{array}{lr} \dot{x}=f(x,u,p), & x(0)=x_{0}\\ y=h(x,p) \end{array} \right. $$

where *x*∈**R**^*n*^ is the state space vector that contains the species included in the biological model under study; *p*∈**R**^*q*^ denotes the parameter vector; *u*∈**R**^*j*^ and *y*∈**R**^*m*^ are the input and output vectors respectively. The key features of this algorithm are:

- simultaneous perturbation of model parameters;
- definition and computation of the evaluation function;
- estimation of the conditional probability density functions (pfds) for each model parameter.

The parameter vector *p* is perturbed through Latin Hypercube sampling (LHS) and the model S is integrated for each one of the *N*_*S*_ samples generated in **R**^*q*^. This procedure allows the collection of *N*_*S*_ vectors of the observables *y*. Then, the CRA is based on the definition and the in silico computation of an evaluation function on a specific output node i.e., on a specific observable *y*_*i*_. In more details, the evaluation function can be formalized as follows:

1 $$\begin{array}{@{}rcl@{}} \zeta:\mathbf{P}\longrightarrow\mathbf{R},\quad z_{i}=\zeta^{S}_{\tau}(p) \end{array} $$

where the index *i* represents the selected output variable of the model. Thus, the evaluation function, that depends on the time behavior of *y*_*i*_ (which, in turn, depends on the selected parameter vector *p*), can be considered as a user defined summary statistic that stands for a specific property of the chosen output node *y*_*i*_. The set of computed evaluation functions, for a specific output node *y*_*i*_, has cardinality equal to *N*_*S*_, i.e. the number of sampled parameter vectors. Let denote with$f_{Z_{i}}(z_{i})$ the pdf of the set of evaluation functions previously defined.

The CRA algorithm aims at quantifying the influence of each model parameter on a specific output node through $f_{Z_{i}}(z_{i})$. In more detail, it is interested in the estimation of the distribution of the parameter vector *p* only when the lower and upper tail of $f_{Z_{i}}(z_{i})$ are selected. To this purpose, the domain of $f_{Z_{i}}(z_{i})$ can be partitioned into two regions by the definition of *L*(*α*) and *U*(*α*) as:

2 $$\begin{array}{@{}rcl@{}} L(\alpha)=\left\{ z_{i} \le a:\int_{0}^{a} f_{Z_{i}}(z_{i}){dz}_{i}=\alpha \right\} \end{array} $$

3 $$\begin{array}{@{}rcl@{}} U(\alpha)=\left\{ z_{i} \ge a:\int_{a}^{\infty} f_{Z_{i}}(z_{i}){dz}_{i}=\alpha \right\} \end{array} $$

where *α* is the level of probability that represents the area under the lower and upper tail of $f_{Z_{i}}(z_{i})$ and *a* is the corresponding threshold value in the domain of the pdf.

The partition defined in the domain of $f_{Z_{i}}(z_{i})$ allows the estimation of two conditional pdfs for each parameter, $f_{p_{i}|L}$ and $f_{p_{i}|U}$, respectively. These two pdfs are the distributions of the parameters of S when the values of the evaluation function belong to the lower and upper tail of $f_{Z_{i}}(z_{i})$ respectively. Here the purpose of the estimation of $f_{p_{i}|L}$ and $f_{p_{i}|U}$ is to select a subset of the *N*_*S*_ samples of the parameters that give rise to the most divergent behaviors of the evaluation function. The two conditional densities defined above are employed for the calculus of the Moment Independent Robustness Indicator (MIRI) according to the following formula:

4 $$\begin{array}{@{}rcl@{}} \mu_{i}=\int |f_{p_{i}|U}-f_{p_{i}|L}|{dp}_{i}, \quad i=1,..., q \end{array} $$

The MIRI is an index that measures the level of separation between $f_{p_{i}|U}$ and $f_{p_{i}|L}$ for each parameter of the model. An high value of the MIRI for a parameter *p*_*i*_ means that the perturbation of the parameter space along the *p*_*i*_ direction leads to high variation of the evaluation function. Thus, the higher the value of a MIRI, the higher is the influence of that parameter on the dynamical behavior of the selected output node.

Finally, the output of CRA is the vector *μ* that contains the value of the MIRI associated to each parameter of the model. For further details about CRA, see [5].

## Implementation

The CRA Toolbox is an open source software developed as a MATLAB package. Its core is a set of functions that the user can run locally in a MATLAB environment by downloading the folder containing the toolbox. This software automates all the functionalities required by CRA to perform robustness analysis of an ODE model.

The CRA Toolbox includes a GUI that runs within MATLAB to encourage the employment of the software also for non expert users. In more detail, the tool firstly performs the import of a mathematical model written in Systems Biology Markup Language (SBML) and saved in *.xml* file format. Then, it allows the user to set the tuning parameters to regulate the parameter space perturbation and the model integration, such as the specific ODE solver to use. Before selecting the reference node from a scrollbar that lists all the outputs of the model, it is necessary to start the simulations by clicking on a specific button. Once the in silico measures are completed, the tool requires the selection of a specific evaluation function in a predefined list and the method for the computation of the lower and higher tail of the pdf of the evaluation function. Finally, it is possible to plot and save in a user defined directory all the in silico measures, the estimated pdfs and the boxplot of MIRIs. In order to guarantee the reliability of results, the toolbox supports the generation of multiple realizations of the entire procedure and of the resulting MIRIs and pdfs. In order to speed up the model simulation we use parallel processing through the Parallel Computing Toolbox ^*TM*^ [11].

Moreover, we also provide an alternative implementation of the CRA Toolbox that allows the user to run the algorithm in batch mode directly from the command line. The core functions and the architecture of the software remains unchanged, but for this version we removed the GUI and we also avoided the use of Simbiology to enhance the portability of the code. Indeed, in this version of the software, the mathematical model can be given in input as a Matlab function where all the ODEs are specified and it is not required to use the SBML language and the corresponding Simbiology Object.

**Fig. 1:**
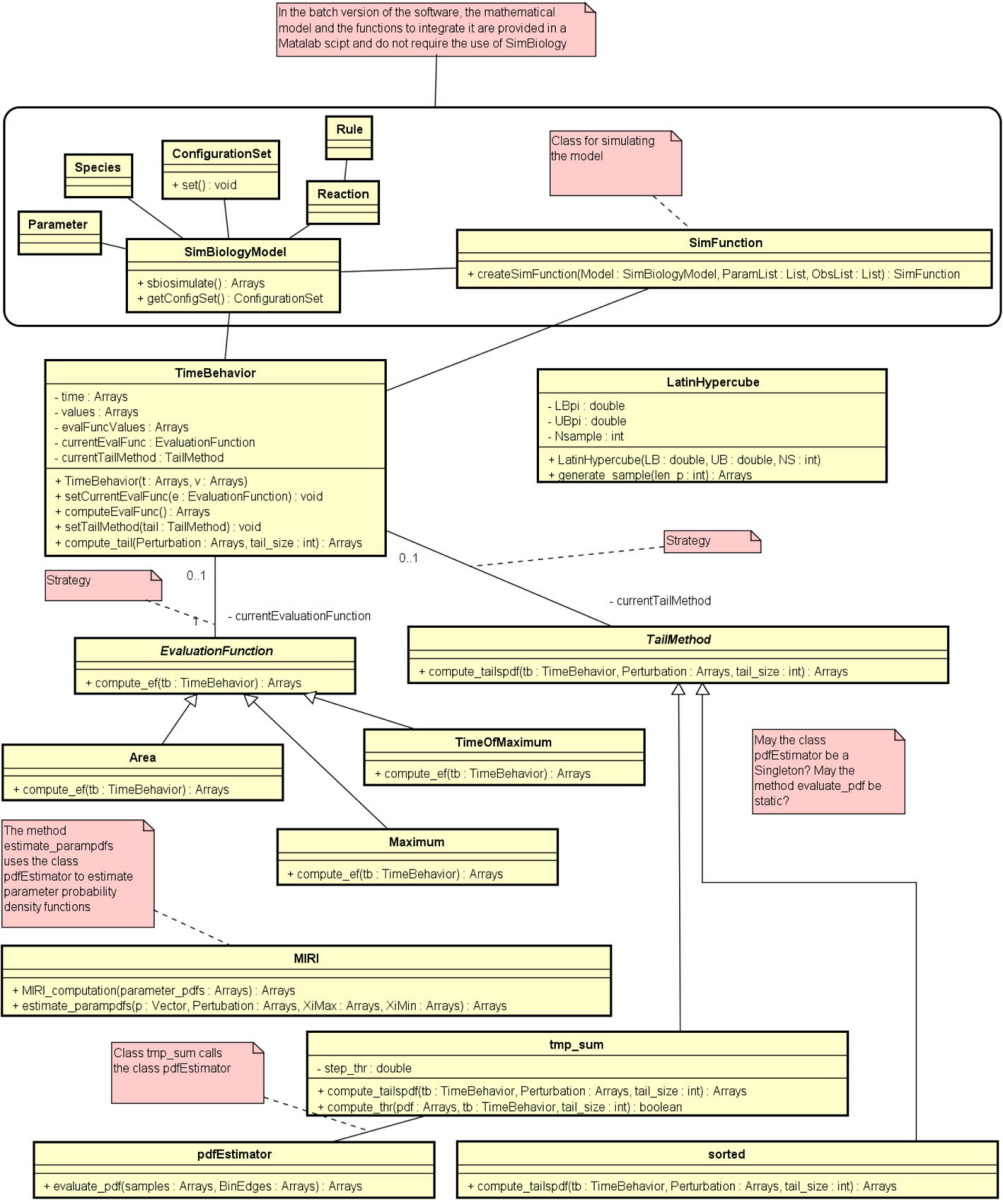
UML class diagram. Classes implemented for the development of the CRA Toolbox. The diagram shows the relations between the different classes, the signatures of the methods and the applied design patterns

The source code of the CRA Toolbox is written according to the Object-Oriented programming paradigm as it is shown in the UML class diagram in Fig. 1. For a detailed description of all the components of the tool see (Additional file 1). The architecture of the software is modular because we implement it using software engineering design patterns to model relationships and interactions between classes. As an example, we use the behavioral Strategy pattern [12] twice between the three main classes of the tool: once between the *TimeBehavior* and *EvaluationFunction* classes and the other between *TimeBehavior* and *Tail* classes. This makes the code easily to extend because if a user wants to add another type of evaluation function or wants to implement another method for the pdf tail calculus, he does not have to change any other part of the code.

## Results

**Fig. 2:**
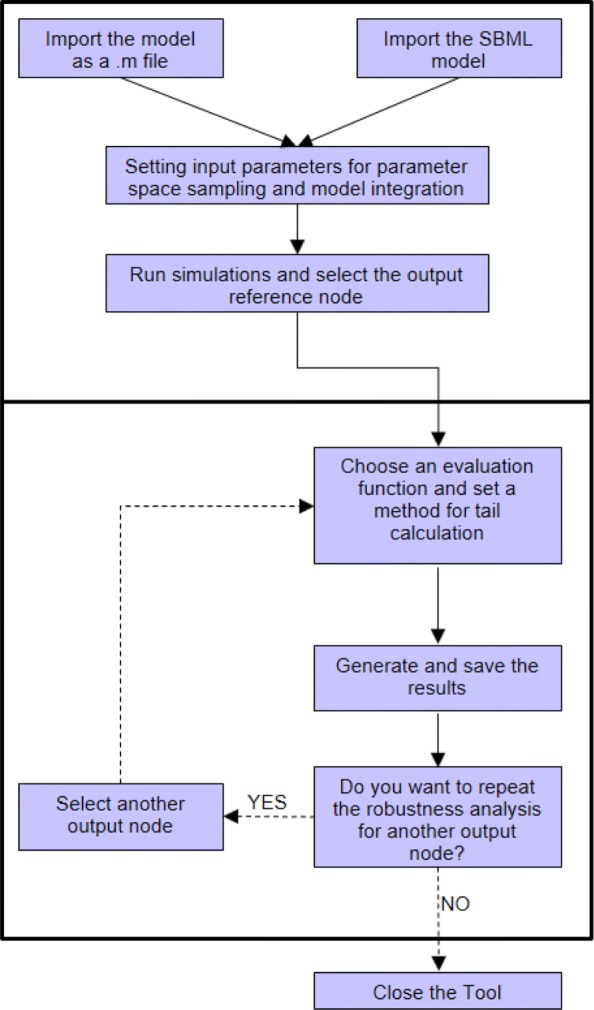
Flowchart of the CRA Toolbox.The flowchart sums up the different steps necessary to perform the CRA

In this section we show how to use the CRA Toolbox and we report the results of the application of CRA to three different ODE models: the Prostate-specific *Pten*^−/−^ mouse model, the Pulse Generator Network and the EGFR-IGF1R pathway. The second and the third examples are used in order to verify that the CRA Toolbox produces results in agreement with those in [5]. Figure 2, Additional files 2 and 3 contain a flowchart and two images to guide the user in the use of the tool.

### Prostate-specific *Pten*^−/−^ mouse model

**Fig. 3:**
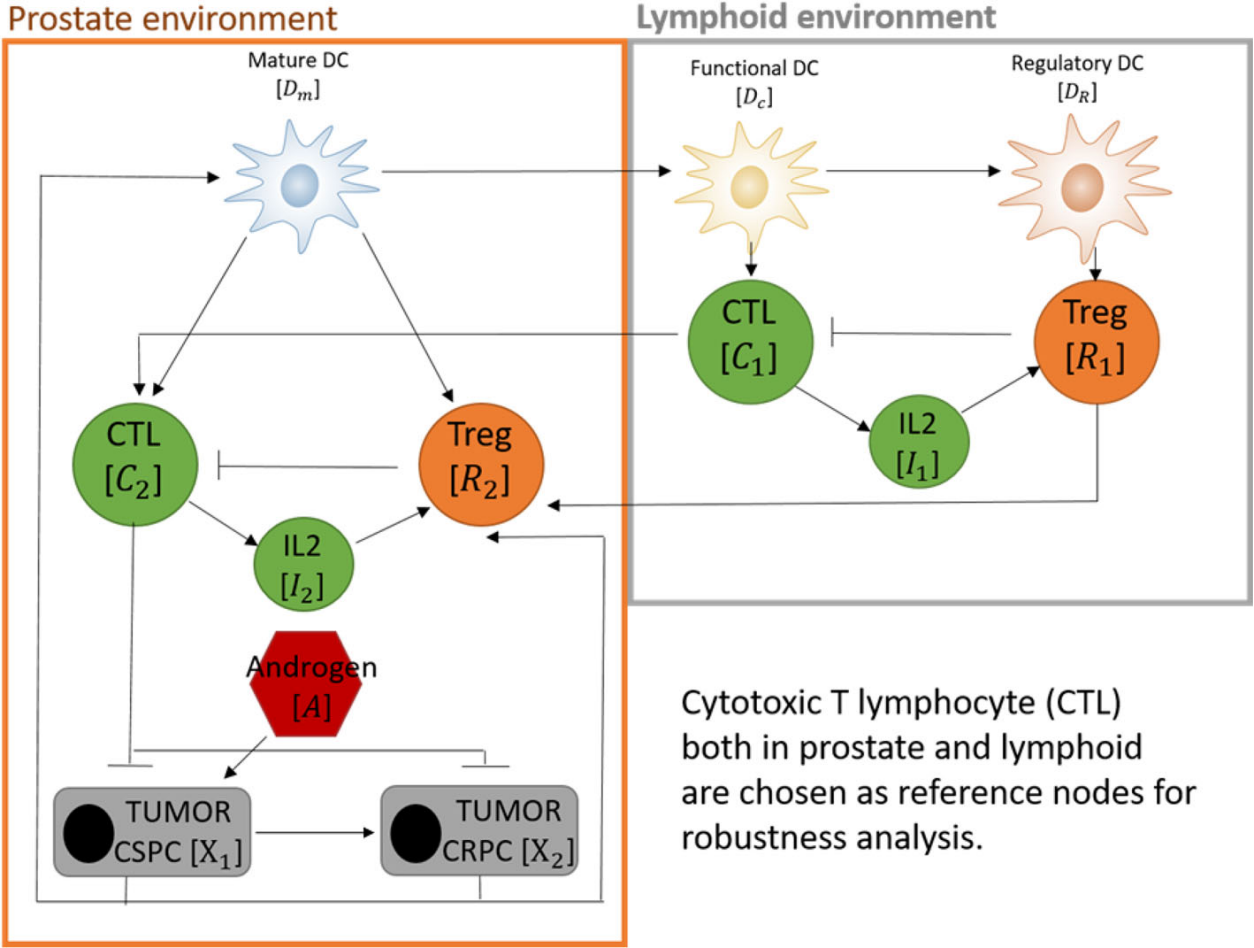
Pathway of the Prostate-specific *Pten*^−/−^ mouse model [13]

In this section, we use the ODE model proposed in [13] to illustrate the functionalities of the CRA Toolbox. This model was developed to study the interactions between prostate cancer and immune microenvironment. In more detail, it is a prostate-specific *Pten*^−/−^ mouse model for analyzing the effect of the combined therapies with vaccines and Androgen deprivation therapy (ADT) in prostate tumor. The original model consists of two compartments, prostate and lymphoid, 11 state variables and four types of therapeutic strategies, resulting in a system of 15 ODEs and 29 parameters. The pathway of the model is shown in Fig. 3. In this example, we analyze a simplified version of this model because we consider only the off-treatment condition (sham-castration). As a result, the mathematical model consists of 12 ODEs and 23 parameters. Equations of the model are:

5 $$\begin{array}{@{}rcl@{}} \left\{\begin{array}{ll} \dot{A}=& \lambda_{A}(1-A) \\ \dot{X_{1}}=& r_{p1}{AX}_{1} - r_{a1}(1-A)X_{1}\\ & - r_{m}(1-A)X_{1} - k_{CX}C_{2}X_{1} \\ \dot{X_{2}}=& r_{p2}X_{2} - r_{a2}X_{2} + r_{m}(1-A)X_{1} - k_{CX}C_{2}X_{2} \\ \dot{D_{m}}=& -\alpha_{XD}(r_{a1}(1-A)X_{1} + k_{CX}C_{2}X_{1} + r_{a2}X_{2}\\ &+ k_{CX}C_{2}X_{2}) - \pi_{D}D_{m} \\ \dot{C_{2}} =&\alpha_{DC}D_{m} + p_{C}\pi_{C}C_{1} - k_{RC}R_{2}C_{2} - \mu_{C}C_{2} \\ \dot{R_{2}} =& \alpha_{DR}D_{m} + p_{R}\pi_{R}R_{1} + \alpha_{IR}I_{2} + \alpha_{XR}(X_{1} + X_{2})\\ & - \mu_{R}R_{2} \\ \dot{I_{2}} =& \alpha_{CI}C_{2} - \mu_{I}I_{2} \\ \dot{D_{C}} =& p_{D}\pi_{D}D_{m} - \alpha_{D_{C}D_{R}}D_{C} \\ \dot{D_{R}} =& \alpha_{D_{C}D_{R}}D_{C} - \mu_{D}D_{R} \\ \dot{C_{1}} =& \alpha_{DC}D_{C} - \mu_{C}C_{1} - k_{RC}R_{1}C_{1} - \pi_{C}C_{1} \\ \dot{R_{1}} =& \alpha_{D_{R}R}D_{R} + \alpha_{IR}I_{1} - \mu_{R}R_{1} - \pi_{R}R_{1} \\ \dot{I_{1}} =& \alpha_{CI}C_{1} - \mu_{I}I_{1} \\ \end{array}\right. \end{array} $$

Parameter values and initial conditions of state variables are shown, respectively, in Tables 1 and 2. Since initial condition of androgen is set to 1, i.e. *A*_0_=1, the concentration of androgen keeps unchanged. Moreover, parameters *p*_*C*_, *p*_*R*_ and *p*_*D*_ represent probabilities fixed all to 0.5 in [13] and thus they are not perturbed in the CRA procedure.

**Table 1:** List of the kinetic parameters of the Prostate-specific *Pten*^−/−^ mouse model and their corresponding nominal values [13]

Parameter name	Value
*λ*_*A*_	0.6931
*r*_*p*1_	0.323432
*r*_*a*1_	0.1
*r*_*m*_	6.702665
*r*_*p*2_	1.426189
*r*_*a*2_	1.061691
*α*_*XD*_	2.096368
*α*_*DC*_	3.077551
*α*_*DR*_	0.739121
*α*_*XR*_	0.144144
*α*_*CI*_	0.408862
*α*_*IR*_	0.354526
$\alpha _{D_{C}D_{R}}$	0.1
$\alpha _{D_{R}R}$	0.4722
*k*_*CX*_	0.1
*k*_*RC*_	1.459617
*π*_*D*_	5.051123
*π*_*C*_	4.111938
*π*_*R*_	0.1
*μ*_*C*_	0.978832
*μ*_*R*_	0.564691
*μ*_*I*_	0.213819
*μ*_*D*_	0.650308

**Table 2:** List of the initial conditions of the Prostate-specific *Pten*^−/−^ mouse model state variables and their corresponding values [13]

State variable	Species	Value
A	Androgen	1
*X*_1_	CSPC	1
*X*_2_	CRPC	0
*D*_*m*_	Mature DC	1
*C*_2_	CTL in prostate	1
*R*_2_	Treg in prostate	1
*I*_2_	IL2 in prostate	1
*D*_*C*_	Functional DC	1
*D*_*R*_	Regulatory DC	1
*C*_1_	CTL in lymphoid	1
*R*_1_	Treg in lymphoid	1
*I*_1_	IL2 in prostate	1

**Fig. 4:**
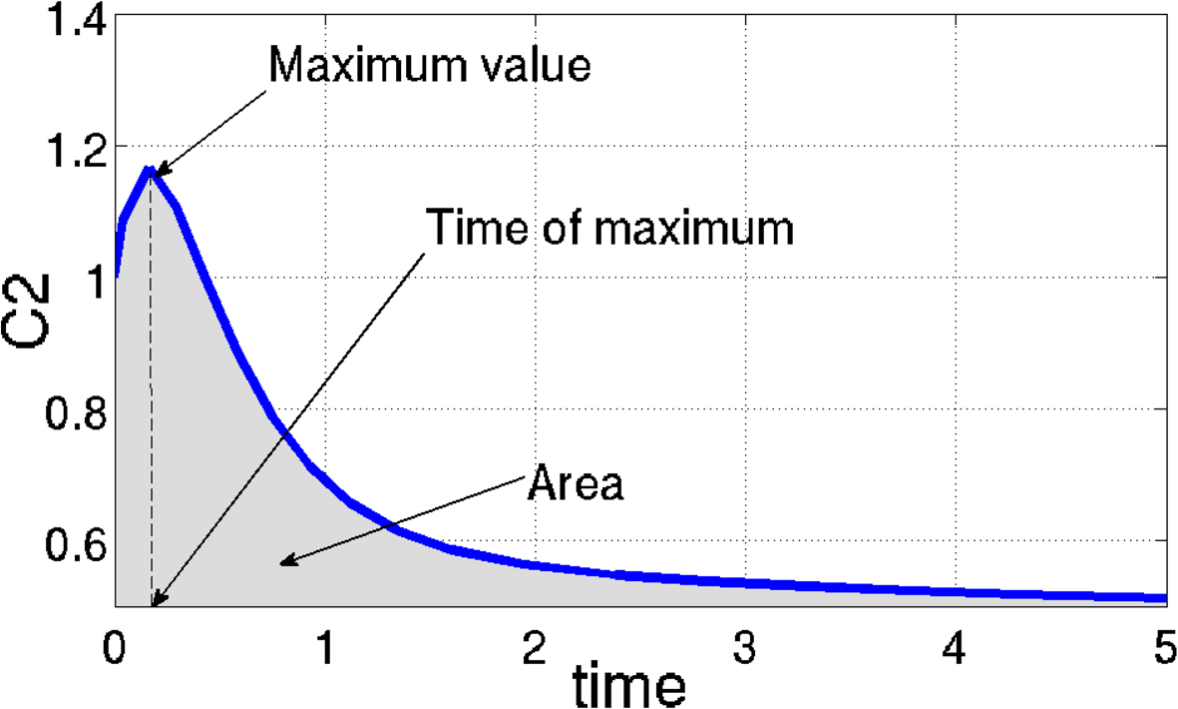
Evaluation functions available in the CRA Toolbox. Nominal time behavior of *C*_2_ (blue line) and the three evaluation functions measured for robustness analysis

We apply the CRA Toolbox to the ODE model by setting tuning parameters of the procedure as follows: number of samples *N*_*S*_ equal to 10000, lower boundary and upper boundary of the LHS equal to 0.1 and 10 respectively. We run 100 independent realizations to verify the reliability and stability of the procedure. We choose different output nodes and evaluation functions in order to show results of the CRA Toolbox in a complete and comprehensive way. Specifically, we select as output nodes both variables *C*_2_ and *C*_1_, which represent cytotoxic T lymphocyte (CTL) in prostate and lymphoid respectively. For *C*_2_, we measure all the three evaluation functions offered by the software, i.e. the area under the curve, the maximum and the time of maximum reached by the time behavior of CTL, as shown in Fig. 4. Conversely, for *C*_1_, we perform robustness analysis using as evaluation function only the area under the curve. In all cases, we set equal to 1000 the dimension of the upper and lower tail of the evaluation function pdf, in order to guarantee a stable estimation of the conditional parameter pdfs [5].

**Fig. 5:**
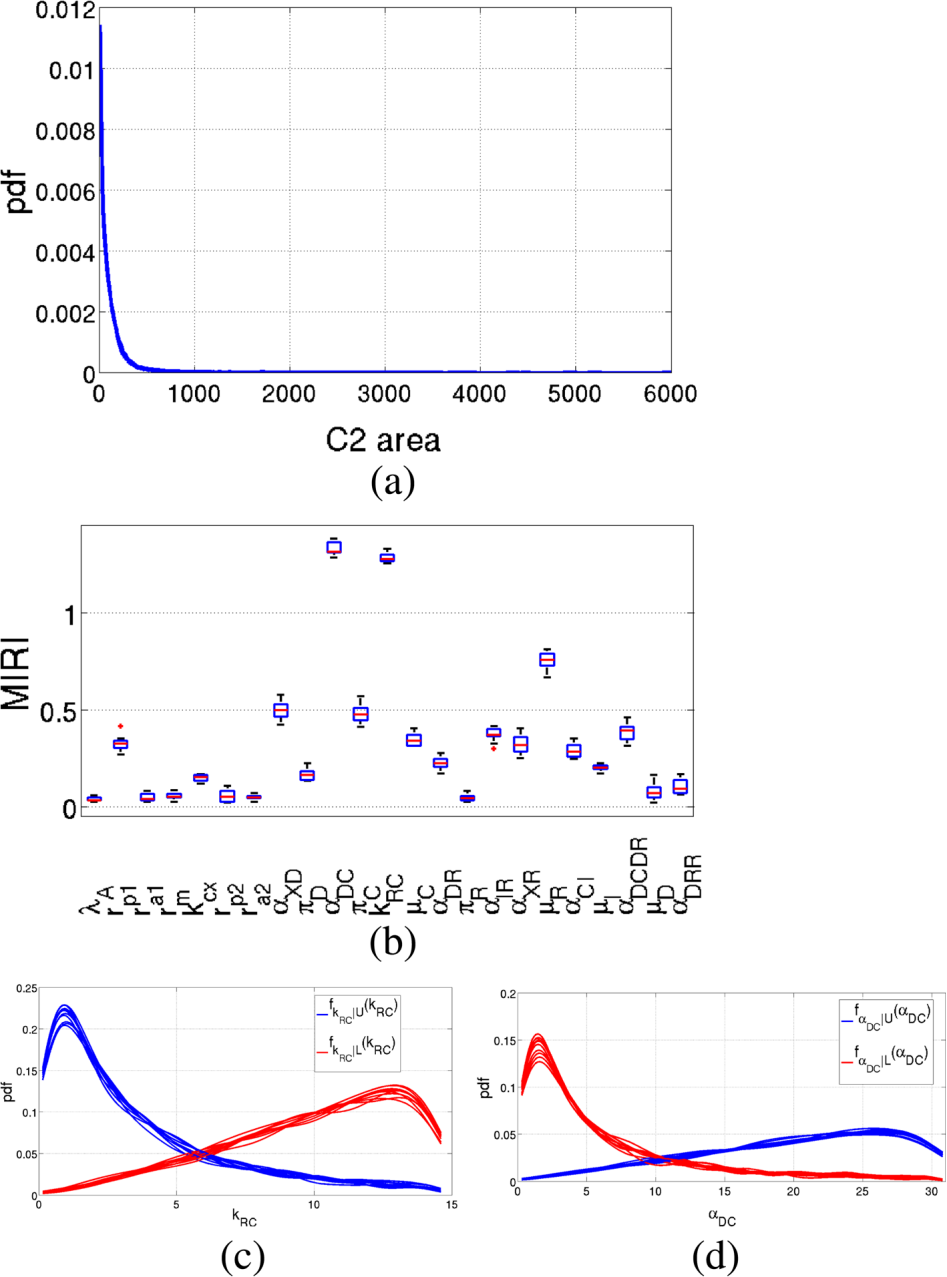
Results of the Prostate-specific *Pten*^−/−^ mouse model: *C*_2_ area. Output of the CRA Toolbox when the area under the curve of *C*_2_ time behavior is chosen as evaluation function. **a** Pdf of the area under the curve of *C*_2_. **b** Boxplot of the 100 realizations of the MIRIs. **c**-**d** Conditional pdfs of the parameters with a MIRI value above 1

Results of CRA applied to variable *C*_2_ to measure the area are shown in Fig. 5. Parameters *α*_*DC*_ and *k*_*RC*_ have MIRI values above 1, thus they have a great impact on the chosen evaluation function. Other parameters influencing the selected output are *μ*_*R*_, *α*_*XD*_ and *π*_*C*_, having MIRI values between 0.5 and 1.

**Fig. 6:**
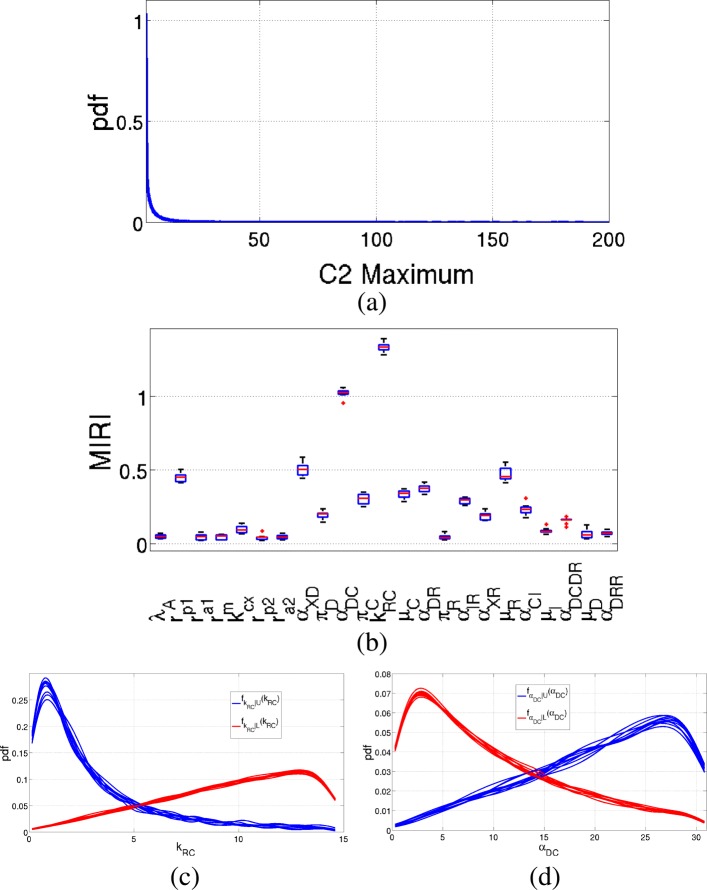
Results of the Prostate-specific *Pten*^−/−^ mouse model: *C*_2_ maximum value. Output of the CRA Toolbox when the maximum value of *C*_2_ time behavior is chosen as evaluation function. **a** Pdf of the maximum value of *C*_2_. **b** Boxplot of the 100 realizations of the MIRIs. **c**-**d** Conditional pdfs of the parameters with a MIRI value above 1

Similar results are obtained for the maximum value of *C*_2_, as shown in Fig. 6. Parameters with the highest MIRI values (∼1) are *k*_*RC*_ and *α*_*DC*_ as before, while parameters *r*_*p*1_, *α*_*XD*_ and *μ*_*R*_ have all MIRIs around 0.5.

**Fig. 7:**
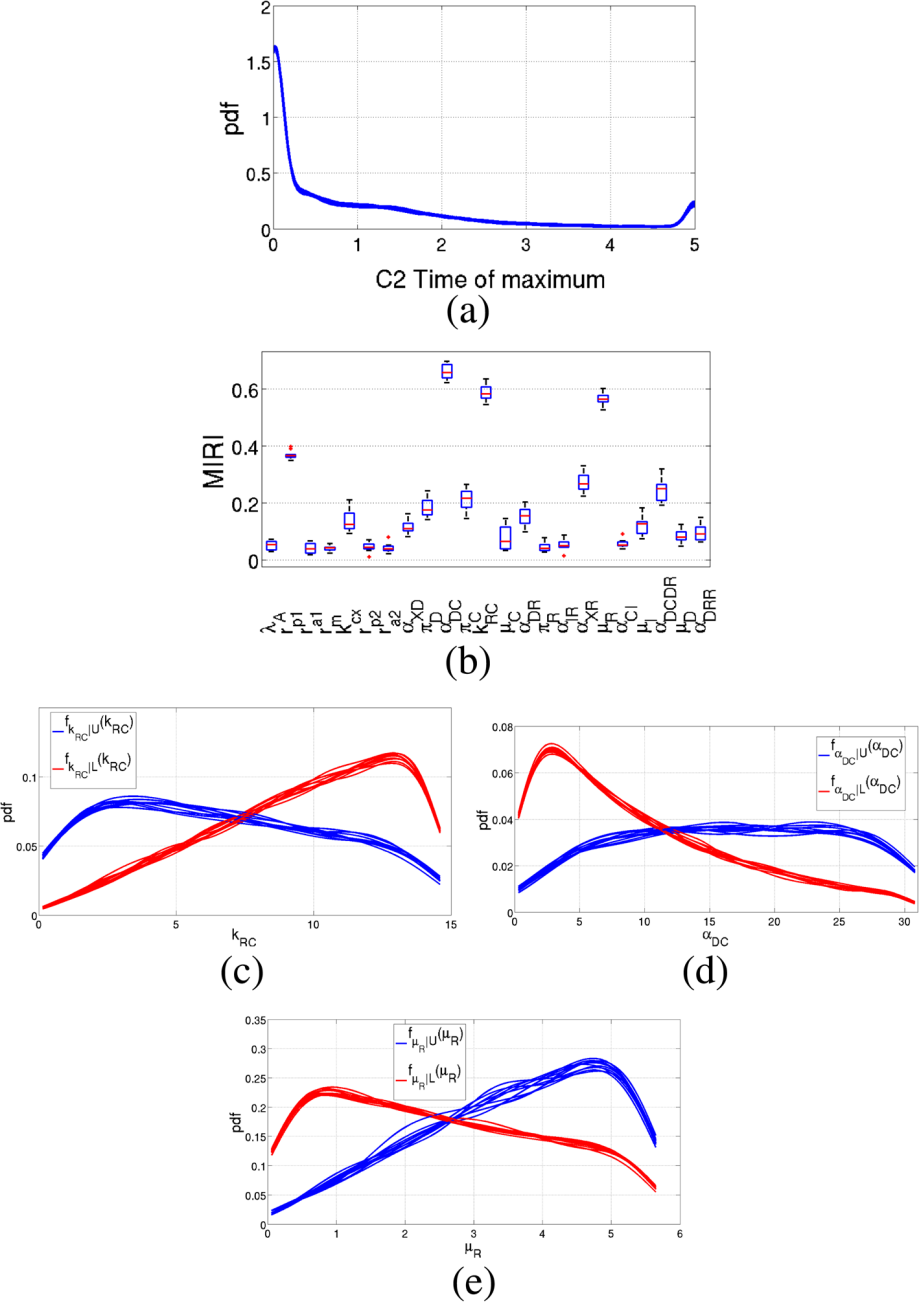
Results of the Prostate-specific *Pten*^−/−^ mouse model: *C*_2_ time of maximum. Output of the CRA Toolbox when the time of maximum of *C*_2_ time behavior is chosen as evaluation function. **a** Pdf of the time of maximum of *C*_2_. **b** Boxplot of the 100 realizations of the MIRIs. **c**-**d**-**e** Conditional pdfs of the parameters with a MIRI value above 0.5

Figure 7 shows results obtained for the time of maximum of variable *C*_2_. In this case, MIRIs have values lower if compared to the previous examples. The most influential parameters are *α*_*DC*_, *k*_*RC*_ and *μ*_*R*_, with MIRI values around 0.6.

**Fig. 8:**
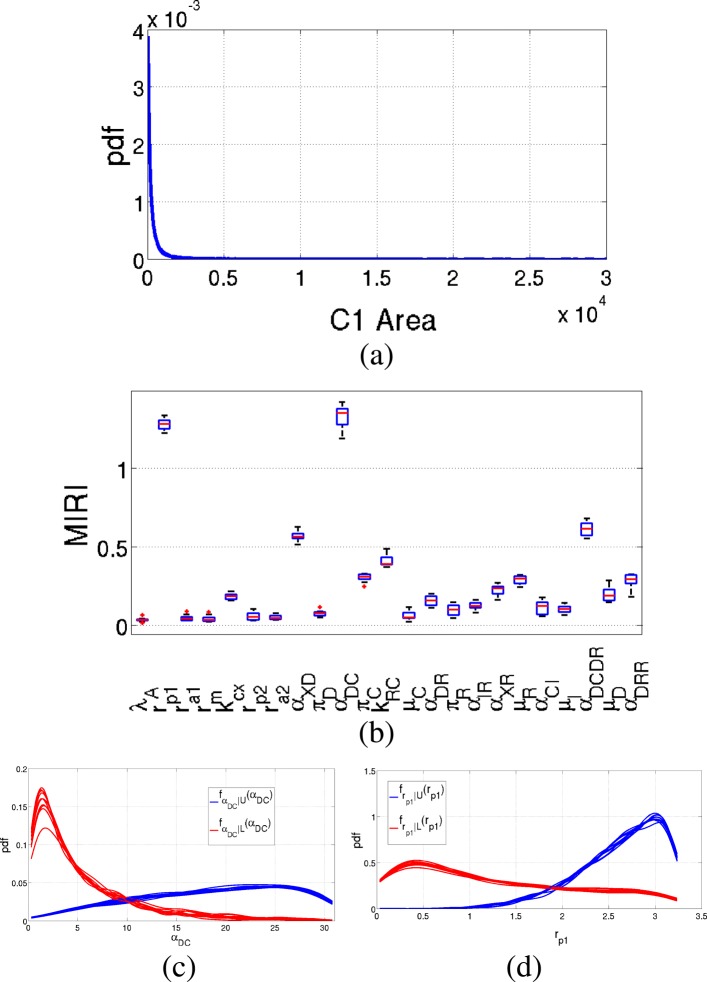
Results of the Prostate-specific *Pten*^−/−^ mouse model: *C*_1_ area. Output of the CRA Toolbox when the area under the curve of *C*_1_ time behavior is chosen as evaluation function. **a** Pdf of the area of *C*_1_. **b** Boxplot of the 100 realizations of the MIRIs. **c**-**d** Conditional pdfs of the parameters with a MIRI value above 1

As regards variable *C*_1_, results of the measured area are shown in Fig. 8. Two are the parameters with MIRIs above 1: *r*_*p*1_ and *α*_*DC*_. All the remaining parameters have low values, except for *α*_*XD*_ and $\alpha _{D_{C}D_{R}}$ with values around 0.5.

Table 5 contains the time necessary to perform all the simulations.

### Pulse generator network

**Fig. 9:**
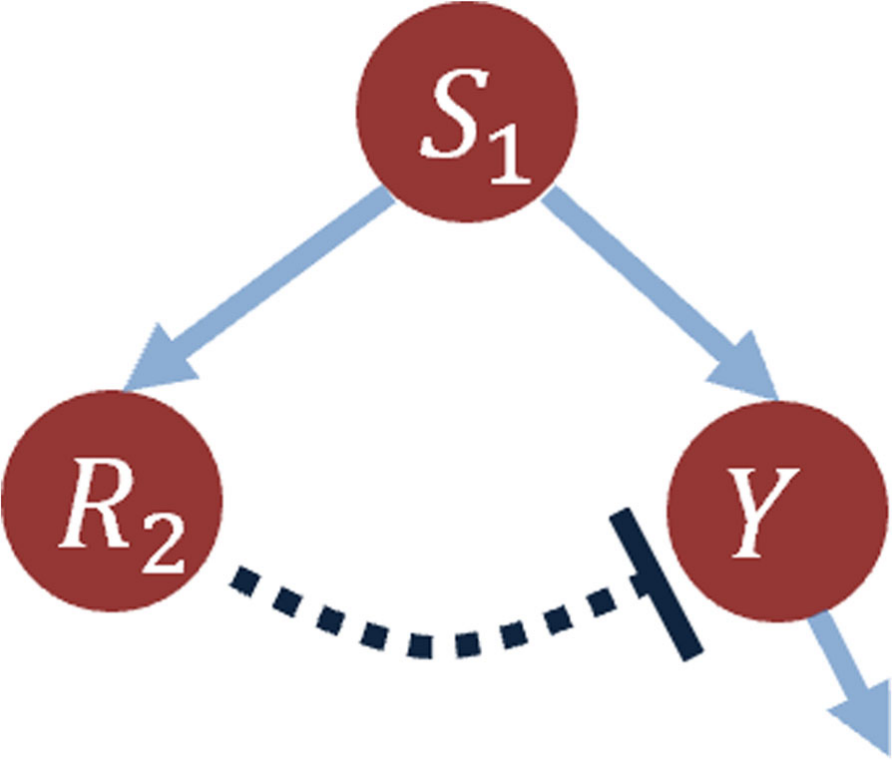
Pulse generator network. Three nodes network of the pulse generator model: solid arrows stand for activation and the T-dashed line represents a deactivation function

We test the CRA Toolbox on a small toy system belonging to the field of Synthetic Biology because synthetic models are one of the best examples of the importance of theoretical modeling in the biological reality [14]. The pulse generator network consists of three nodes, representing three genes, aimed at producing a transient output response to a persistent input stimulus [14]. Figure 9 shows the interaction schema of the model. Node S1 is the input step signal that activates both R2 and Y. R2 is the so called repressor because it acts as a deactivator of the product Y. The corresponding ODE model has two state variables, eight kinetic parameters and one input signal. The following ODEs are written using a Hill function for the activation and repression function [5, 15]:

6 $$\begin{array}{@{}rcl@{}}  \left\{\begin{array}{ll} &\dot{R_{2}}=k_{1}\frac{(S_{1}/K_{1})^{n_{1}}}{1+(S_{1}/K_{1})^{n_{1}}}-\lambda_{2}R_{2}\\ &\dot{Y}=\frac{k_{12}}{1+(R_{2}/K_{2})^{n_{2}}}\frac{(S_{1}/K_{1})^{n_{1}}}{1+(S_{1}/K_{1})^{n_{1}}}-\lambda Y. \end{array}\right. \end{array} $$

**Fig. 10:**
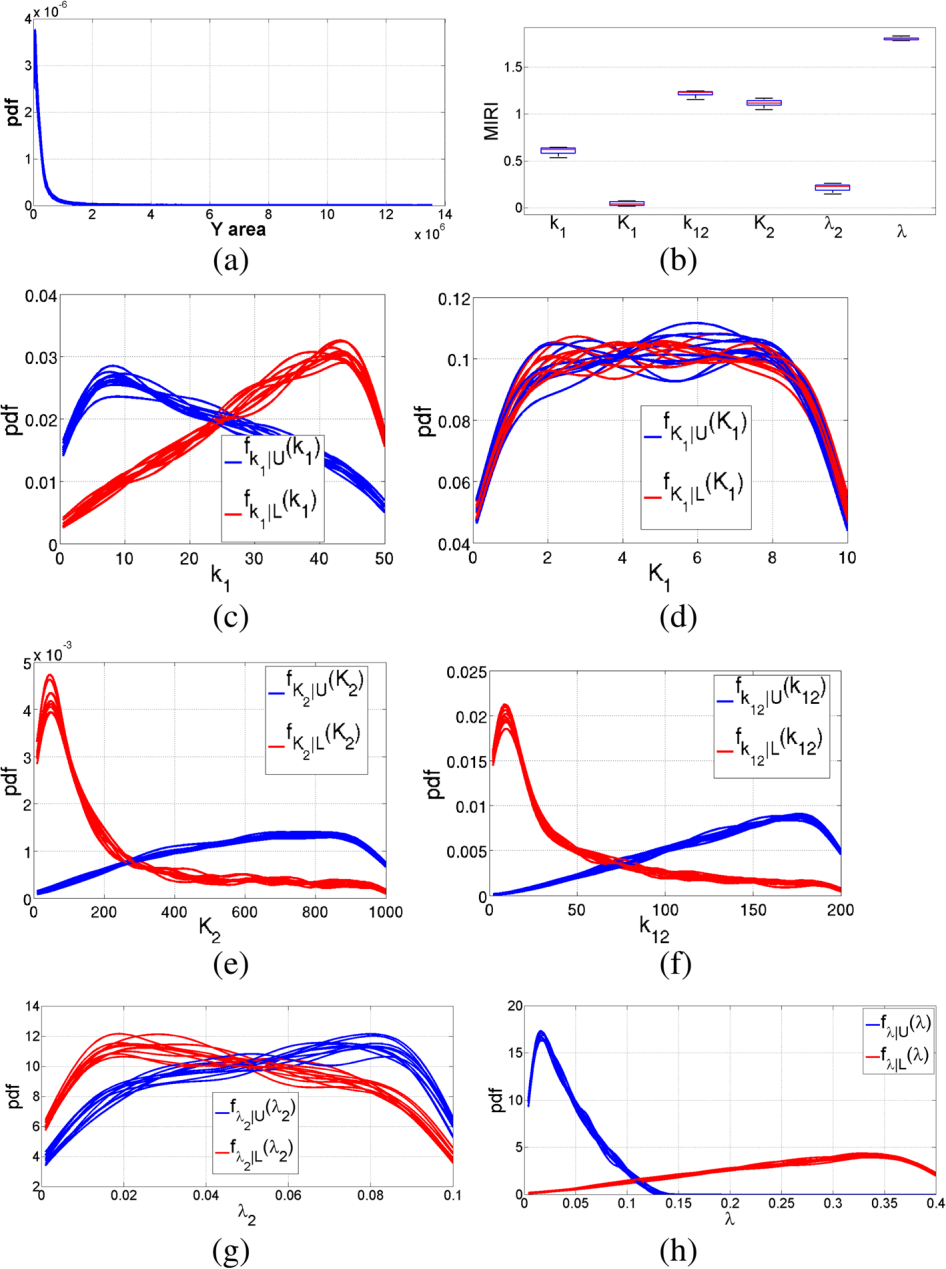
Pulse generator network results: Y area. Output of the CRA Toolbox selecting the area of Y as evaluation function. **a** Pdf of the area of Y. **b** Boxplot of the MIRIs for the 100 realizations. **c**-**d**-**e**-**f**-**g**-**h** Conditional pdfs of parameters *k*_1_, *K*_1_, *K*_2_, *k*_12_, *λ*_2_ and *λ* respectively

**Fig. 11:**
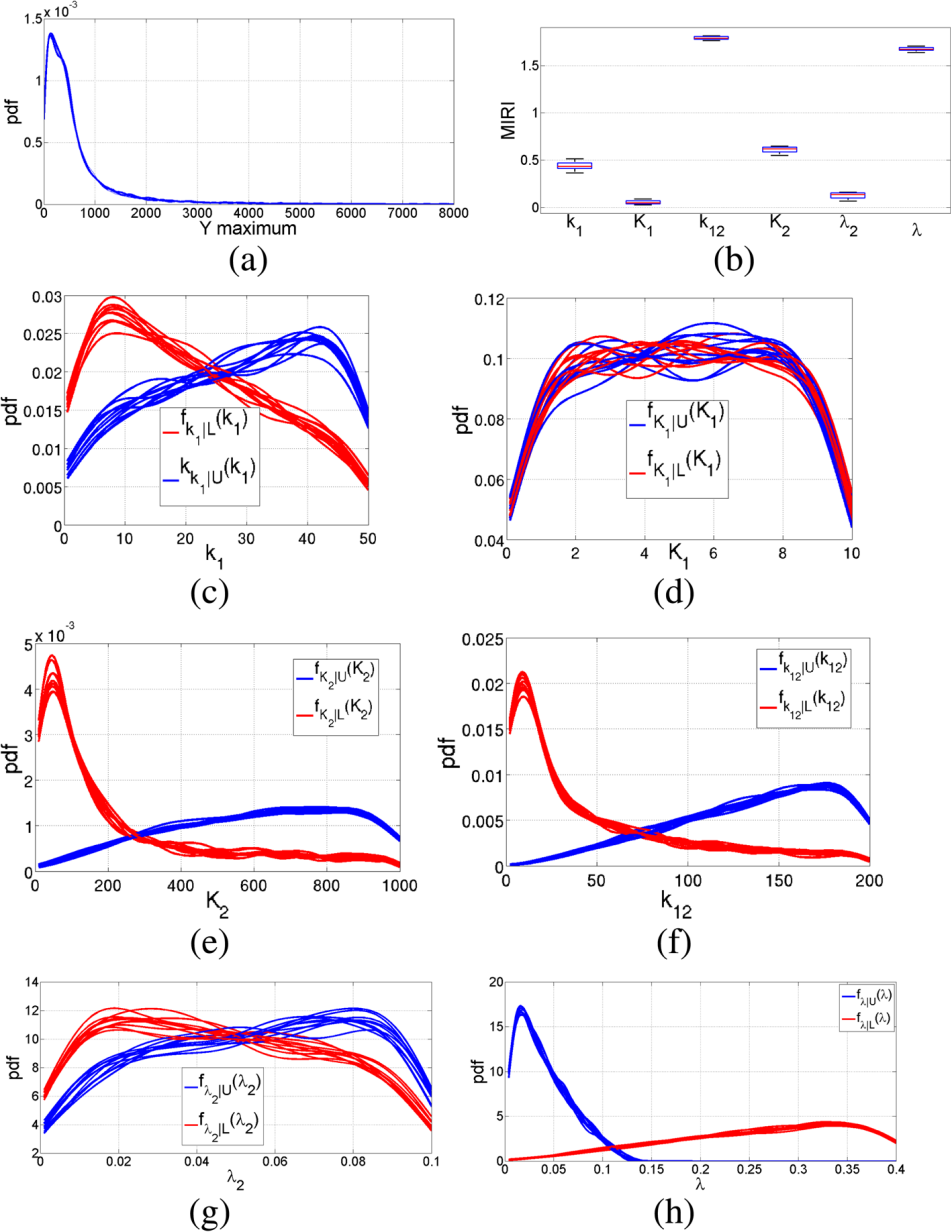
Pulse generator network results: maximum of Y. Output of the CRA Toolbox selecting the maximum of Y as evaluation function. **a** Pdf of the maximum of Y. **b** Boxplot of the MIRIs for the 100 realizations. **c**-**d**-**e**-**f**-**g**-**h** Conditional pdfs of parameters *k*_1_, *K*_1_, *K*_2_, *k*_12_, *λ*_2_ and *λ* respectively

**Fig. 12:**
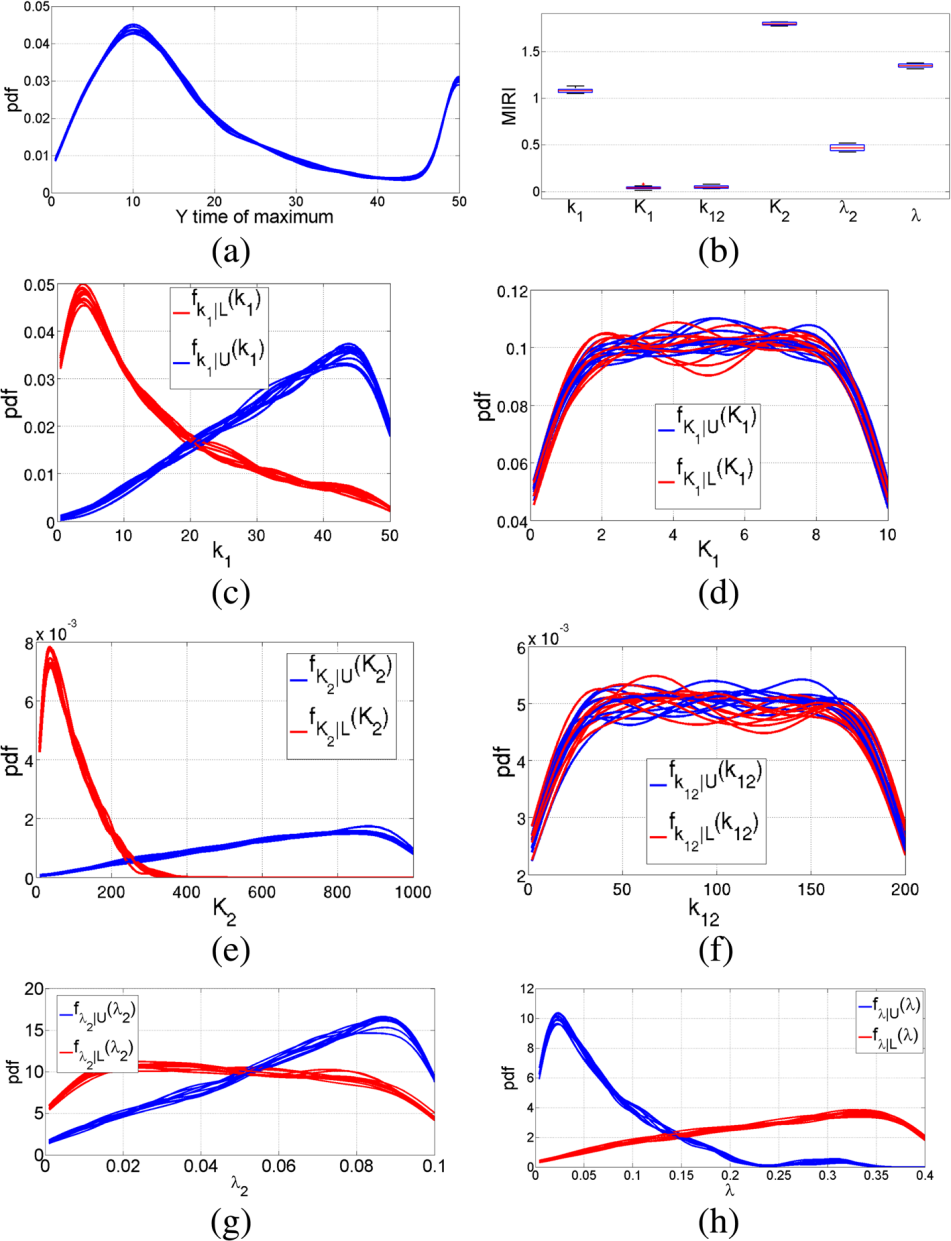
Pulse generator network results: time of maximum of Y. Output of the CRA Toolbox selecting the time of maximum of Y as evaluation function. **a** Pdf of the time of maximum of Y. **b** Boxplot of the MIRIs for the 100 realizations. **c**-**d**-**e**-**f**-**g**-**h** Conditional pdfs of parameters *k*_1_, *K*_1_, *K*_2_, *k*_12_, *λ*_2_ and *λ* respectively

Nominal values for the parameters in Eq. 6 are *k*_1_=5 nM/min, *k*_12_=20 nM/min, *λ*_2_=0.01 nM/min, *λ*=0.04 nM/min, *K*_1_=1 nM, *K*_2_=100 nM and *n*_1_= *n*_2_=3. We run the CRA Toolbox setting the tuning parameters as reported in [5]. More in detail, we set the number of realizations, the lower boundary, the upper boundary and the number of samples equal to 100, 0.1, 10 and 10000 respectively. Parameters *n*_1_ and *n*_2_ remain fixed to their nominal values since they are not included in robustness analysis. We select as reference node the observable Y and we progressively perform the CRA using all the three evaluation functions provided by the tool, i.e. the area under the curve, the maximum value and the time of maximum. Figure 10 shows the pdf of the area and the corresponding boxplot of MIRIs when choosing 1000 as the dimension of both the lower and higher tails. The other subfigures of Fig. 10 show the conditional pdfs of each parameter that are used for the calculus of MIRIs. Figure 11 shows the results of robustness analysis when the maximum of output variable Y is selected as evaluation function. Figure 12 reports the output generated by the CRA Toolbox about the time of maximum of variable Y. From Figs. 10b, 11b and 12b, it is clear how parameter *λ* has a great impact on the output behavior of Y, since it influences all the three evaluation functions with an high value of the corresponding MIRI. Moreover, parameter *k*_12_ is the one with highest value of the MIRI in Fig. 11b and thus it especially influences the maximum value reached by the Y node. Finally, parameter *K*_2_ is the most relevant when dealing with the time of maximum of variable Y because it has the highest MIRI value in Fig. 12b. Table 5 reports the time required to complete all the simulations described in this section.

### EGFR-IGF1R pathway in lung cancer

**Fig. 13:**
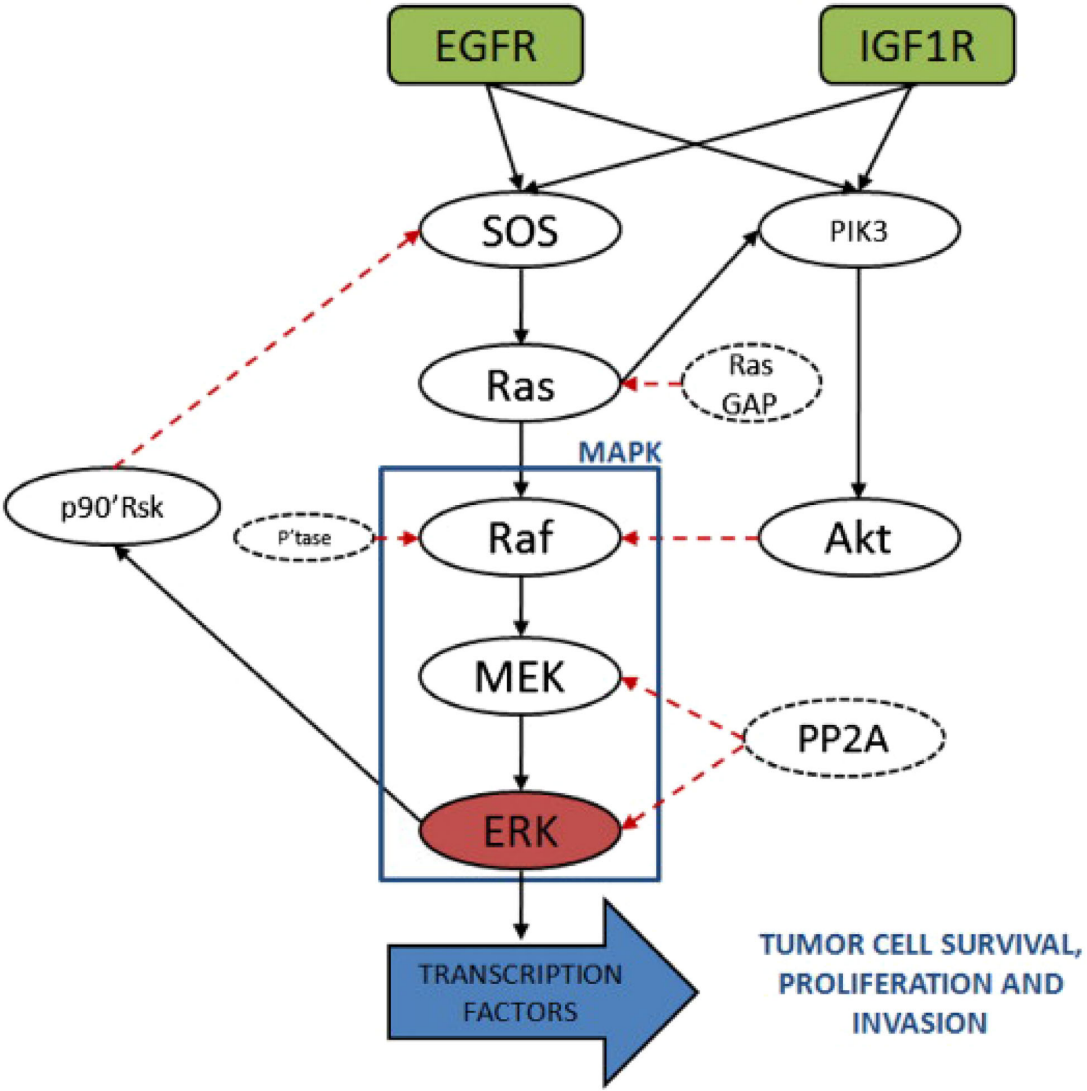
Pathway of the EGFR-IGF1R model in lung cancer [16]

The last example of application of the CRA Toolbox is the EGFR-IGF1R pathway, which is one of the most relevant interaction network for the study of cancer pathogenesis and progression in Non-Small Cell Lung Cancer (NSCLC). Figure 13 depicts the pathway. For detailed information about the biological importance of this network and the role of the different nodes see [16]. The corresponding mathematical ODE model is published in [17] (id:MODEL1209230000). The dynamical ODE model is composed of ten equations (Eq. 7), containing two types of kinetic laws: the law of mass action and the Michaelis-Menten kinetics.

7 $$\begin{array}{@{}rcl@{}}  \left\{\begin{array}{ll} \dot{x_{1}}=&-p_{1}x_{1}\\ \dot{x_{2}}=&-p_{2}x_{2}\\ \dot{x_{3}}=&\ p_{6}x_{1}\frac{x_{3}^{T}-x_{3}}{p_{7}+x_{3}^{T}-x_{3}} + p_{14}x_{2}\frac{x_{3}^{T}-x_{3}}{p_{15}+x_{3}^{T}-x_{3}}\\ &-p_{12}x_{8}\frac{x_{3}}{p_{13}+x_{3}}\\ \dot{x_{4}}=&\ p_{8}x_{3}\frac{x_{4}^{T}-x_{4}}{p_{9}+x_{4}^{T}-x_{4}} - p_{33}u_{3}\frac{x_{4}}{p_{34}+x_{4}}\\ \dot{x_{5}}=&\ p_{27}x_{4}\frac{{x_{5}}^{T}-x_{5}}{p_{28}+{x_{5}}^{T}-x_{5}} - p_{37}u_{1}\frac{x_{5}}{p_{38}+x_{5}}\\ &-p_{31}x_{10}\frac{x_{5}}{p_{32}+x_{5}}\\ \dot{x_{6}}=&\ p_{29}x_{5}\frac{{x_{6}}^{T}-x_{6}}{p_{30}+{x_{6}}^{T}-x_{6}}-p_{35}u_{2}\frac{x_{6}}{p_{36}+x_{6}}\\ \dot{x_{7}}=&\ p_{10}x_{6}\frac{{x_{7}}^{T}-x_{7}}{p_{11}+{x_{7}}^{T}-x_{7}}-p_{23}u_{2}\frac{x_{7}}{p_{24}+x_{7}} \\ \dot{x_{8}}=&\ p_{4}x_{7}\frac{x_{8}^{T}-x_{8}}{p_{5}+{x_{8}}^{T}-x_{8}}-p_{39}x_{8}\\ \dot{x_{9}}=&\ p_{25}x_{4}\frac{x_{9}^{T}-x_{9}}{p_{26}+x_{9}^{T}-x_{9}}+p_{16}x_{2}\frac{x_{9}^{T}-x_{9}}{p_{17}+{x_{9}^{T}-x_{9}}}\\ &+p_{18}x_{1}\frac{x_{9}^{T}-x_{9}}{p_{19}+{x_{9}}^{T}-x_{9}}-p_{3}x_{9}\\ \dot{x_{10}}=&\ p_{20}x_{9}\frac{x_{10}^{T}-x_{10}}{p_{21}+x_{10}^{T}-x_{10}}-p_{22}x_{10} \end{array}\right. \end{array} $$

**Fig. 14:**
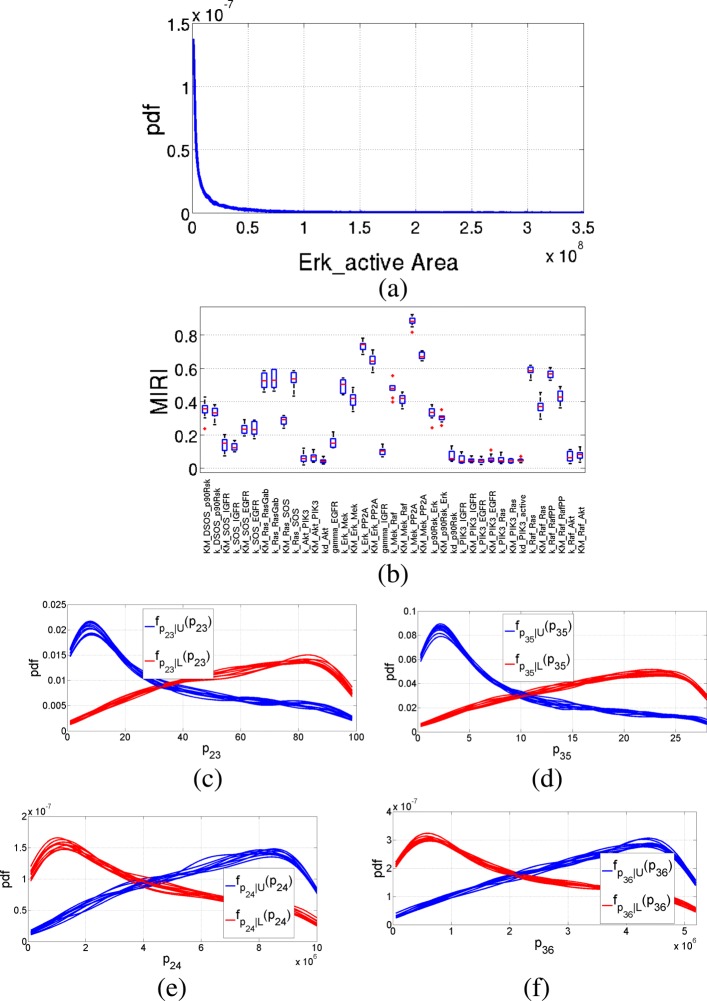
EGFR-IGF1R pathway results: ERK area. Output of the CRA Toolbox selecting as reference node ERK. **a** Pdf of the area under the curve of ERK. **b** Boxplot of the 100 realizations of the MIRIs. **c**-**d**-**e**-**f** Conditional pdfs of the parameters with a MIRI value above 0.6

Table 3 explicits names and initial concentrations of the ten state variables, of the three input signals and of the eight total protein parameters. Table 4 lists all the kinetic model parameters and their nominal values. We run the CRA Toolbox according to the guidelines in [5]: the number of realizations, the lower boundary, the upper boundary and the number of samples are set equal to 100, 0.1, 10 and 10000 respectively. We select as reference node the active form of ERK protein, since it is one of the best indicator of the proliferation attitude of lung cancer. More in detail, we perform robustness analysis of the model choosing as evaluation function the area under the curve of the time behavior of ERK. Figure 14a shows the pdf of the evaluation function and Fig. 14b the boxplot for each one of the 39 MIRI parameters. This figure clearly shows that four parameters have a MIRI higher than 0.6 and thus they significantly influence the dynamical behavior of the output node. More in detail, these parameters are: *k*_*ERK-PP2A*_, *KM*_*ERK-PP2A*_, *k*_*MEK-PP2A*_ and *KM*_*MEK-PP2A*_ and Fig. 14c, d, e and f show the corresponding conditional pdfs.

**Table 3:** List of species included in the EGFR-IGF1R model and the corresponding initial concentrations and total protein amount

State Variable	Species	Value
*x*_1_	*EGFR* ^∗^	8000
*x*_2_	*IGF1R* ^∗^	650
*x*_3_	*SOS*	0
*x*_4_	*Ras* ^∗^	0
*x*_5_	*Raf* ^∗^	0
*x*_6_	*MEK* ^∗^	0
*x*_7_	*Erk* ^∗^	0
*x*_8_	*p90Rsk* ^∗^	0
*x*_9_	*PIK3* ^∗^	0
*x*_10_	*Akt* ^∗^	0
*u*_1_	*RafPP*	120000
*u*_2_	*PP2A*	120000
*u*_3_	*RasGab*	120000
$x_{3}^{T}$	*DSOS*	120000
$x_{4}^{T}$	*Ras*	120000
$x_{5}^{T}$	*Raf*	120000
$x_{6}^{T}$	*MEK*	600000
$x_{7}^{T}$	*Erk*	600000
$x_{8}^{T}$	*p90Rsk*	120000
$x_{9}^{T}$	*PIK3*	120000
$x_{10}^{T}$	*Akt*	120000

**Table 4:** List of the kinetic parameters of the EGFR-IGF1R model and their corresponding nominal values

Parameter	Name	Value
*p*_1_	*γ*_*EGFR*_	0.02
*p*_2_	*γ*_*IGF1R*_	0.02
*p*_3_	*k* *d*_*PIK3*_	0.005
*p*_4_	*k*_*p90Rsk-Erk*_	0.0213697
*p*_5_	*K* *M*_*p90Rsk-Erk*_	763523
*p*_6_	*k*_*SOS-EGFR*_	694.731
*p*_7_	*KM*_*SOS-EGFR*_	6086070
*p*_8_	*k*_*Ras-SOS*_	32.344
*p*_9_	*KM*_*Ras-SOS*_	35954.3
*p*_10_	*k*_*Erk-MEK*_	9.85367
*p*_11_	*KM*_*Erk-MEK*_	1007340
*p*_12_	*k*_*DSOS-p90Rsk*_	161197
*p*_13_	*KM*_*DSOS-p90Rsk*_	896896
*p*_14_	*k*_*SOS-IGFR*_	500
*p*_15_	*KM*_*SOS-IGFR*_	1000000
*p*_16_	*k*_*PIK3-IGF1R*_	10.6737
*p*_17_	*KM*_*PIK3-IGF1R*_	184912
*p*_18_	*k*_*PIK3-EGFR*_	10.6737
*p*_19_	*KM*_*PIK3-EGFR*_	184912
*p*_20_	*k*_*Akt-PIK3*_	0.0566279
*p*_21_	*KM*_*Akt-PIK3*_	653951
*p*_22_	*kd*_*Akt*_	0.005
*p*_23_	*k*_*Erk-PP2A*_	9.85367
*p*_24_	*KM*_*Erk-PP2A*_	1007340
*p*_25_	*k*_*PIK3-Ras*_	0.0771067
*p*_26_	*KM*_*PIK3-Ras*_	272056
*p*_27_	*k*_*Raf-Ras*_	0.884096
*p*_28_	*KM*_*Raf-Ras*_	62464.6
*p*_29_	*k*_*Raf-MEK*_	185.759
*p*_30_	*KM*_*Raf-MEK*_	4768350
*p*_31_	*k*_*Raf-Akt*_	15.1212
*p*_32_	*KM*_*Raf-Akt*_	119355
*p*_33_	*k*_*Ras-RasGab*_	1509.36
*p*_34_	*KM*_*Ras-RasGab*_	1432410
*p*_35_	*k*_*MEK-PP2A*_	2.83243
*p*_36_	*KM*_*MEK-PP2A*_	518753
*p*_37_	*k*_*Raf-RafPP*_	0.126329
*p*_38_	*KM*_*Raf-RafPP*_	1061.71
*p*_39_	*kd*_*p90Rsk*_	0.005

Table 5 reports the time required to complete the robustness analysis through the CRA algorithm.

**Table 5:** Computational cost to run the CRA algorithm in all the examples provided in the Results section

Model	Output node	Evaluation Function	Time (sec.)
		Area	45
Pulse Generator Network	Y	Maximum	45
		Time of Maximum	42
		Area	1702
	C2	Maximum	1703
Prostate-specific *Pten*^−/−^ mouse model		Time of Maximum	1706
	C1	Area	1708
EGFR-IGF1R pathway in lung cancer	ERK	Area	102

The times are expressed in seconds and they refer to a single realization of the CRA

## Discussion

The CRA is an algorithm to study the robustness of complex biological networks and it allows the identification of few parameters that have a major impact on the behavior of a selected output variable. One of its main innovations is the introduction of a sensitivity measure, the MIRI, that takes advantage of all the conditional distributions of the parameters, without reference to a specific moment. In [18] there are the mathematical details of this class of indicators and the comparison with the variance-based uncertainty importance measures.

Here we present the CRA Toolbox, a software package for MATLAB aimed at performing the robustness analysis based on the paradigm of the CRA. The tool consists of a set of MATLAB functions that automate all the necessary mathematical steps from the integration of an ODE model until the calculus of the MIRIs. We decide to use the Object-Oriented programming paradigm because it allows us the development of an extendable and flexible architecture through the implementation of different engineering design patterns. In this way, other users can add blocks of software to define novel evaluation functions and other methods for the identification of the pdf tails without modifying the structure of the software and the source code of the existing classes.

Here we tested the CRA on three ODE models that contain different kinetic laws and different number of state variables. All the simulations were performed on a Intel Core i7-4700HQ CPU, 2.40GHz 8, 16-GB memory, Ubuntu 16.04 LTS (64bit). From Table 5 it is clear how the time to complete a realization of the CRA highly depends both on the dimension of the model and on the number of samples *N*_*S*_ of the LHS. Indeed, from a computational point of view, the most intensive part of the algorithm is the integration of the mathematical model *N*_*S*_ times. Moreover, the choice of *N*_*S*_ influences the estimation of the evaluation function pdf, whose cost increases with *N*_*S*_. On the other hand, the computation of the MIRIs is pretty fast, independently of the type of evaluation function selected. For more details about the choice of the tuning parameters and the computational costs of the CRA for different settings of tuning parameters see Additional file 4.

One of the key ideas in [5] is that the theoretical statements of the CRA do not depend on the specific modeling technique used to represent a biological phenomenon. For this reason, one of the possible future development of the tool is to augment the number and types of formats of mathematical models taken in input. Finally, the code of the CRA Toolbox can be easily adapted to other open source programming languages such as Octave and Python.

## Conclusions

The CRA Toolbox is unique in the category of the robustness analysis tools because it is the specific implementation of the CRA with all its features. It has the scope to enlarge and facilitate the usage of this algorithm and to disclosure it also to non expert users. This can significantly help the oncological research of physicians in discover novel targeted therapies. Moreover, in [19], the Conditional Robustness Calibration (CRC) algorithm is presented, which is an upgrade of the CRA that allows the generation of MIRIs for a given model taking into account multiple nodes simultaneously. This novel algorithm suggests that the CRA Toolbox can be modified and extended in the future in order to implement also the functionalities of CRC.

## Availability data and requirements

**Project name:** CRA Toolbox

**Project home page:** http://gitlab.ict4life.com/SysBiOThe/CRA-Matlab

**Operating system(s):** Platform independent

**Programming language:** MATLAB

**Other requirements:** MATLAB

**License:** MATLAB

**Any restrictions to use by non-academics:** License needed for MATLAB

## Additional files

Additional file 1 This *.pdf* file is a detailed description of all the classes and methods implemented in the tool and it is a useful guide for users to understand how to run and use the software package. (PDF 62 kb)

Additional file 2 This *.png* file is a screenshot of the first part of the GUI of the CRA Toolbox. (PNG 47 kb)

Additional file 3 This *.png* file is a screenshot of the second part of the GUI of the CRA Toolbox. (PNG 60 kb)

Additional file 4 This *.pdf* file is the user guide of the CRA Toolbox. (PDF 327 kb)

## Data Availability

The most recent version of the software and the documentation can be found in a public available GitLab repository using the following link: http://gitlab.ict4life.com/SysBiOThe/CRA-Matlab.
