# Supplementary material for: CRA toolbox: software package for conditional robustness analysis of cancer systems biology models in MATLAB
Source: BMC Bioinformatics. 2019 Jul 9;20:385. doi: 10.1186/s12859-019-2933-z (PMC6617887; doi:10.1186/s12859-019-2933-z)
Supplement: Supplementary file 1 — This .pdf file is a detailed description of all the classes and methods implemented in the tool and it is a useful guide for users to understand how to run and use the software package. (PDF 62 kb) [file 12859_2019_2933_MOESM1_ESM.pdf]

#################################
Files description
#################################
BEFORE RUNNING THE TOOLBOX, THE USER MUST ADD TO THE PATH THE FOLDER CLASSES AND THE FOLDER EXAMPLES WHERE THE MODELS ARE SAVED

This is the main code folder for running the CRA Toolbox with a Matlab GUI and a model written in SBML Language. It contains the following files:

1. gui_CRA.m is the function for starting the main GUI. It contains all the functions of the elements in the main GUI.
   It recalls the following functions:
   1.1 start_simulation.m: function that executes the CRA algorithm.
2. gui_CRA.fig is the figure of the graphical interface defined in gui_CRA.m. It allows us to specify the following parameters for
   performing the CRA:
   - stop time of the simulation;
   - type of ode solver and step of the time vector;
   - number of independent realizations of the procedure to perform;
   - lower and upper boundaries of the Latin Hypercube to generate;
   - number of samples of the Latin Hypercube;
   - variable of the model to set as reference node to measure.
3. gui_MIRI.m is the function for starting the second GUI for computing MIRI. It contains all the callbacks of the elements in the GUI.
   It recalls the following functions:
   3.1 compute_MIRI.m: function that computes MIRI of the model parameters with respect to the chosen output variable
   3.2 plotpdf_param.m: function for visualizing the conditional probability density function (pdf) of all
   the model parameters.
4. gui_MIRI.fig is the figure of the graphical interface defined in gui_MIRI.m. It allows us to specify the following parameters for
   computing MIRI of the output node selected in the previous GUI:
   - type of evaluation function. It is possible to choose among three evaluation functions: area under the curve, maximum value and time
     of maximum of the time behavior of the chosen variable;
   - number of samples N to include in the upper and lower tail of the evaluation function pdf;
   - method for computing the tails of the evaluation function pdf. It is possible to choose between two methods: sort and tmp_sum.
     The sort method orders all the samples of the evaluation function and selects the N lowest and highest values.
     The tmp_sum method calculates the lower and upper quartile of the pdf, computing the threshold values in an adaptive manner.
     The initial lower threshold is set to 0 and it is repeatedly increased of the step size defined by the user until at least N samples
     are included in the tail. The same procedure is repeated for the upper tail, starting from a threshold equal to 1.
5. start_simulation.m is the function executed when the button START SIMULATION in the main GUI is pushed. It takes in input
   the handles of the main GUI in order to retrieve all the variables inserted by the user. For each independent realization,
   it generates the Latin Hypercube and simulates the model.
6. compute_MIRI.m is the function executed when the button COMPUTE MIRI in the second GUI (gui_MIRI) is pushed. It takes in input

the handles of both GUIs and it computes MIRI of the selected variable. It plots boxplot of MIRI values and the pdf of the evaluation function of the chosen variable. It saves MIRI values and the pdfs of both the evaluation function and parameters of the model. It also generates the histogram of the evaluation function.

7. plotpdf_evalfunc.m is the function that plots and saves the estimated pdf of the evaluation function for the chosen output node.
8. plotpdf_param.m is the function that plots and saves the conditional pdfs of model parameters.

The folder Classes contains the following classes necessary for running the CRA Toolbox:

1. EvaluationFunction.m is the abstract class for a generic evaluation function.
2. Area.m is a concrete class that extends the abstract class EvaluationFunction. It computes the area under the curve of a model variable.
3. Maximum.m is a concrete class that extends the abstract class EvaluationFunction. It computes the maximum value of a variable curve
4. TimeOfMaximum.m is a concrete class that extends the abstract class EvaluationFunction. It computes the maximum point value of a variable curve.
5. TailMethod.m is the abstract class for defining a generic method that computes upper and lower tails of a parameter pdf. The upper tail is conditioned to high values of the evaluation function while the lower tail is conditioned to low values of the evaluation function.
6. sorted.m is a concrete class that extends the abstract class TailMethod.
7. tmp_sum.m is a concrete class that extends the abstract class TailMethod.
8. TimeBehavior.m is the class representing the time behavior of a model variable.
9. LatinHypercube.m is the class representing the Latin Hypercube generated for perturbing the parameter space.
10. pdfEstimator.m is the class representing an object for estimating a probability density function through the method ksdensity
11. MIRI.m is the class for computing the Moment Independent Robustness Indicator (MIRI) of each model parameter

The folder Examples contains some ODE models in SBML format:

- PulseGenerator.xml
- EGFR_IG1FRmodel.xml
- model_Peng2016_reduced.xml
