# Supplementary figures and images for: CRA toolbox: software package for conditional robustness analysis of cancer systems biology models in MATLAB
Source: BMC Bioinformatics. 2019 Jul 9;20:385. doi: 10.1186/s12859-019-2933-z (PMC6617887; doi:10.1186/s12859-019-2933-z)

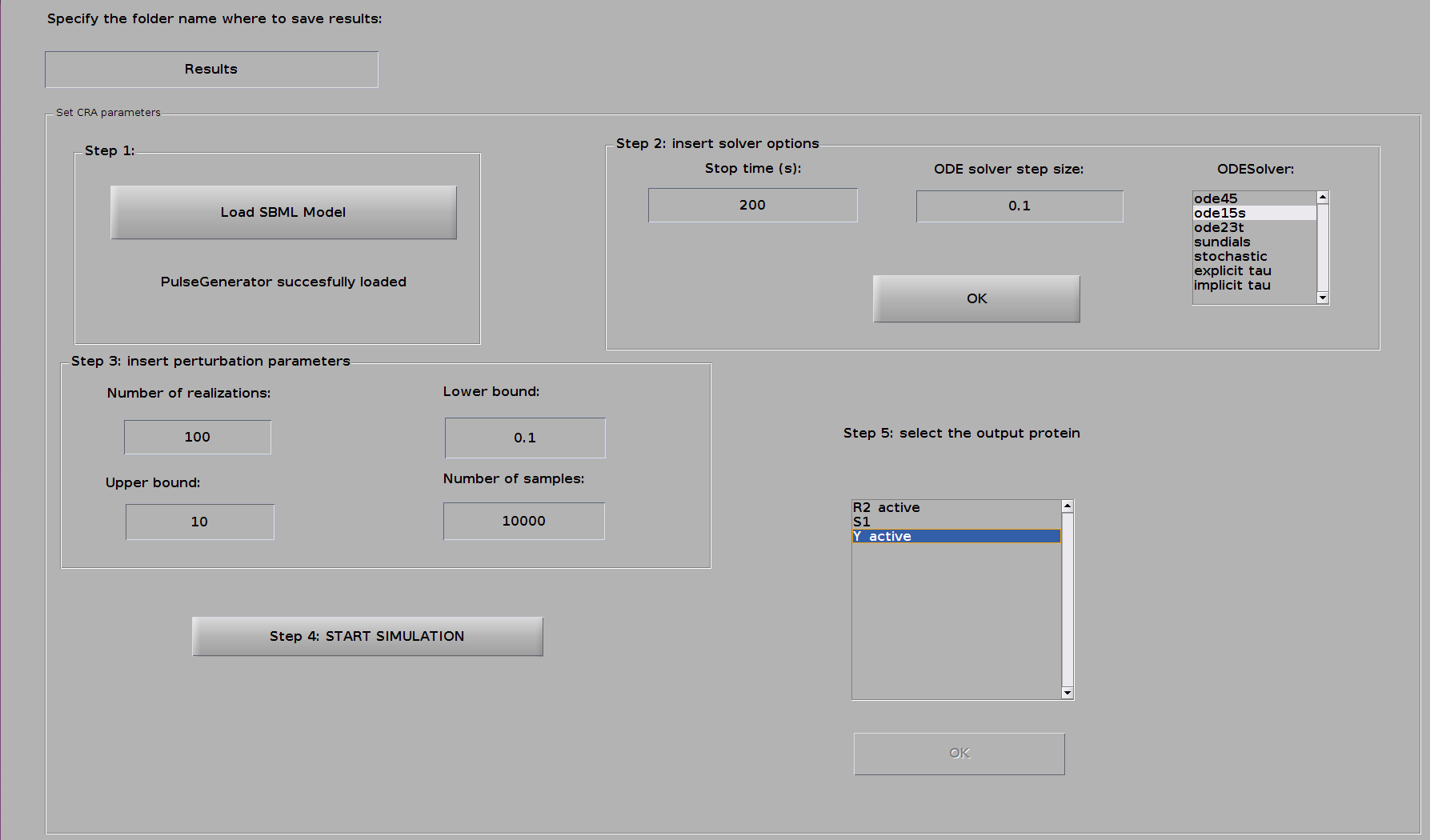

Supplement: Supplementary file 2 — This .png file is a screenshot of the first part of the GUI of the CRA Toolbox. (PNG 47 kb) [file 12859_2019_2933_MOESM2_ESM.png]

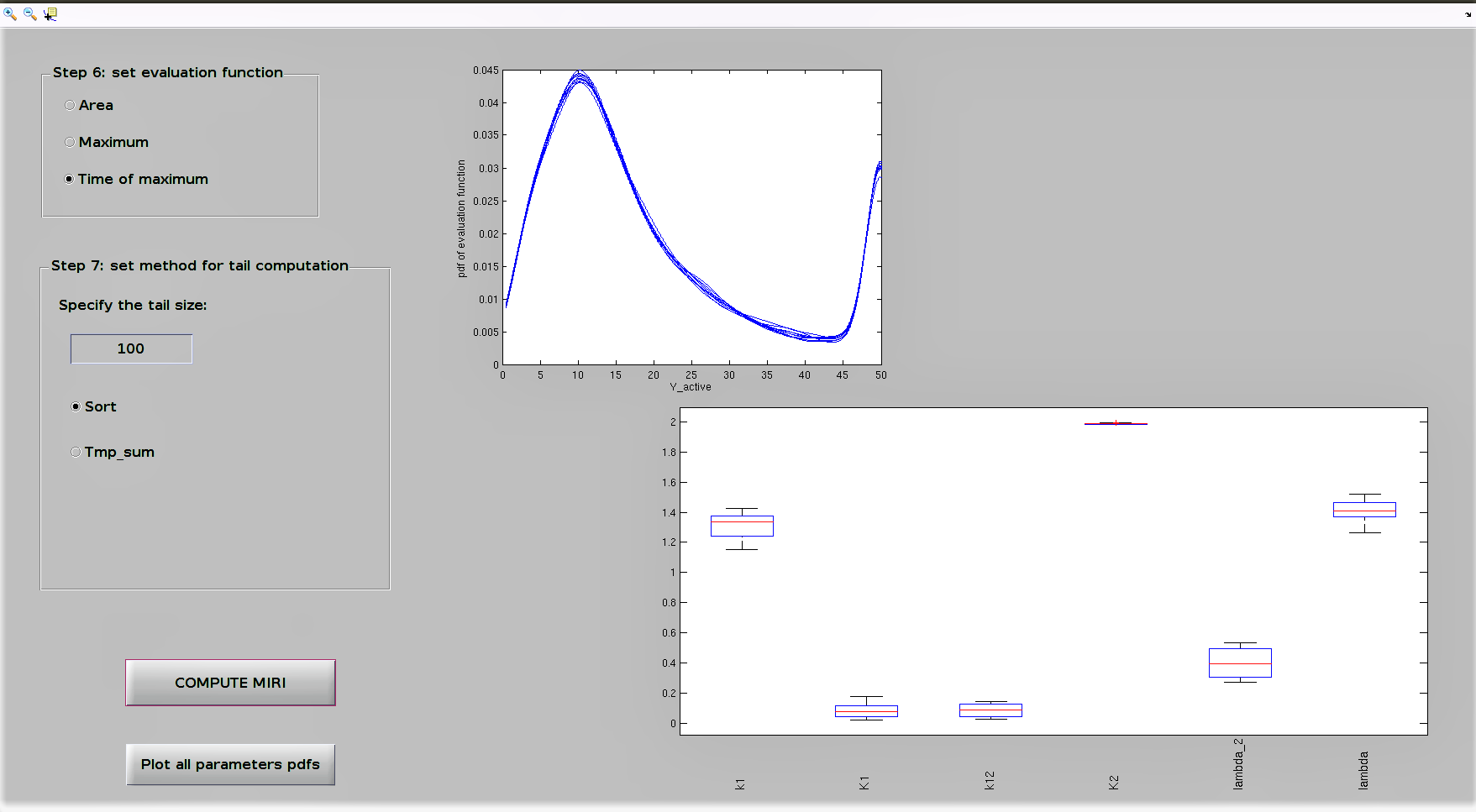

Supplement: Supplementary file 3 — This .png file is a screenshot of the second part of the GUI of the CRA Toolbox. (PNG 60 kb) [file 12859_2019_2933_MOESM3_ESM.png]
