# Supplementary material for: CRA toolbox: software package for conditional robustness analysis of cancer systems biology models in MATLAB
Source: BMC Bioinformatics. 2019 Jul 9;20:385. doi: 10.1186/s12859-019-2933-z (PMC6617887; doi:10.1186/s12859-019-2933-z)
Supplement: Supplementary file 4 — This .pdf file is the user guide of the CRA Toolbox. (PDF 327 kb) [file 12859_2019_2933_MOESM4_ESM.pdf]

CRA Toolbox

F.Bianconi, C.Antonini, L.Tomassoni, P.Valigi

March 29, 2019

1 Introduction

The CRA Toolbox is a set of MATLAB functions for performing conditional robustness analysis of Ordinary Differential Equation (ODE) models. The model can be written in Systems Biology Markup Language (SBML) or as a MATLAB function where all the equations of the observables are specified. Moreover the CRA Toolbox can be run using a Graphical User Interface (GUI), especially suitable for the non expert users, or through a main script where all the input parameters are specified. The algorithm can do the following things:

1. Perturb the parameter space of an ODE model using Latin Hypercube Sampling (LHS);
2. simulate the ODE model using all the generated parameter vectors;
3. selection and computation of the evaluation function representative of the property of interest in the model;
4. computation of the Moment Independent Robustness Indicator (MIRI) for each model parameter, in order to evaluate how each parameter influences the behavior of the evaluation function;
5. do plots of MIRIs and probability density functions (pdfs) of the evaluation function and the parameters.

Download

The full Matlab code and the documentation are available from `http://gitlab.ict4life.com/SysBiOThe/CRA-Matlab`. To install the toolbox clone the folder CRA-Matlab to a specific directory by running:

```
git clone http://gitlab.ict4life.com/SysBiOThe/CRA-Matlab.git
```

or by downloading the zip file directly from `http://gitlab.ict4life.com/SysBiOThe/CRA-Matlab/tree/master`.

MATLAB code

The code can be run using a Graphical User Interface (GUI) where the model input has to be provided in Systems Biology Markup Language (SBML) and it is then imported using the SimBiology Toolbox. Alternatively, it is possible

to run the bash version of the CRA where the mathematical model can be specified as a MATLAB function, where all the model equations are reported (see Examples).
The main functions of the Toolbox are:

- *start_simulation.m* which simulates the ODE model using as parameter vectors those generated through the LHS. The user needs to specify the number of samples and the interval of variation of the parameters.

- *compute_MIRI.m*. For each parameter vector generated by the LHS, the function computes the evaluation function chosen by the user and the MIRI for each model parameter.

- *plotpdf_evalfunc.m* which calculates and shows the pdf of the evaluation function given the perturbed parameter space using the *ksdensity* function.

- *plotpdf_param.m* which estimates the conditional upper and lower densities of each parameter through the *ksdensity* function.

Example: Pulse Generator Network

This example is a synthetic system of three genes receiving a persistent input stimulus. It is taken from Szallasi, Zoltan, Jrg Stelling, and Vipul Periwal. "System modeling in cellular biology." From Concepts to (2006).

$$\begin{aligned} \dot{R_2} &= k_1 \frac{(S_1/K_1)^{n_1}}{1+(S_1/K_1)^{n_1}} - \lambda_2 R_2 \\ \dot{Y} &= \frac{k_{12}}{1+(R_2/K_2)^{n_2}} \frac{(S_1/K_1)^{n_1}}{1+(S_1/K_1)^{n_1}} - \lambda Y. \end{aligned} \tag{2}$$

The nominal parameter values of the equations are k_1=5 nM/min, k_{12}=20 nM/min, λ_2=0.01 nM/min, λ=0.04 nM/min, K_1=1 nM, K_2=100 nM and n_1=n_2=3 and the input signal is S_1=470 nM. When using the bash version of the algorithm, the first thing to do is to write a function that describes the mathematical model:

```
function dx=PulseGeneratorNetwork(t,x,p,u,xT)
%names of model parameters
k1=p(1);
K1=p(2);
lambda2=p(3);
k12=p(4);
K2=p(5);
lambda=p(6);
%input signal
S1=u(1);
%state variables
R2=x(1);
Y=x(2);
%ODEs
dR2 = k1*((S1/K1)^3)/(1+(S1/K1)^3) - lambda2*R2;
dY = k12/(1+(R2/K2)^3)*((S1/K1)^3/(1+(S1/K1)^3)) -
    lambda*Y;
dx=[dR2;dY];
end
```

Next, in the main script, specify the name of the function where the model is defined:

```
model_name='PulseGeneratorNetwork';
```

Then, define the time axis for model simulation:

```
stop_time=200;
step_size=0.5;
time_axis=[0:step_size:stop_time]';
```

Define the type of ode solver for integrating the model:

```
ode_solver='ode15s';
```

Define nominal parameter values of the model and their names, initial conditions of state variables, input signals. Moreover, also the number and name of observables, i.e. those state variables that are in the output vector, are defined:

```
%parameters and initial conditions of the model
nominal_parameters=[5 1 0.01 20 100 0.04];
parameters_name={'k1','K1','lambda2','k12','K2','
    lambda'};
x0=[0 0];
u=470;
num_observables=2;
observables_name={'R1','Y'};
```

A struct is created to store all the model parameters reported above:

```
model=struct('name',model_name,'odesolver',ode_solver,
    'time',time_axis,'stop',stop_time,'step',step_size,
    'nominal_parameters',nominal_parameters,'
    parameters_name',{parameters_name},'num_observables
    ',num_observables,'observables_name',{
    observables_name},'initial_conditions',x0,'input',u
    ,'total_proteins',0);
```

Then, once the model is defined, the number of independent realizations of the algorithm to perform and the folder where all the results will be stored have to be specified:

```
Nr=10;
folder='retesintetica_test2';
```

Here, we define the lower and upper boundaries of the Latin Hypercube Sampling (LHS) and the number of samples to generate.

```
LBpi=0.1; %lower boundary
UBpi=10;  %upper boundary
Ns=10000;  %number of samples
```

Then the name of the output variable and its property to be measured are specified. Currently, it is possible to choose among three evaluation functions: area under the curve, maximum value and time of maximum for the time behavior of the selected variable. The user can also define his own evaluation function in a .m file by extending the abstract class EvaluationFunction

```
variable_name='Y';
current_func=Area(); %current evaluation function
```

Here, we specify the number of samples to include in the upper and lower tail when computing the pdf of the evaluation function and the method for computing the tails of the pdf of the evaluation function. Right now, it is possible to choose between two methods: *sorted*() which sorts the values of the evaluation function and selects the first and last samples according to *tail_size*; *tmp_sum*() computes the tails by selecting the upper and lower quartile of the evaluation function. When using *tmp_sum*(), the parameter *step_size* needs also to be specified. It is the step for computing the lower and upper quartile of the pdf in an iterative way. Indeed, when the upper and lower tails do not have the number of samples specified by the user in *tail_size*, the threshold is increased of a quantity equal to *step_size* and the calculation of the tails is repeated. The user can also define his own method for the tails computation by extending the abstract class TailMethod().

```
tail_size=1000;  %number of samples for the lower and
    upper tail
step_size=0.01;
current_tm=tmp_sum(step_size));
```

This is the function for simulating the model using the perturbed parameter vectors generated through LHS. The function returns the time behavior of all output variables and the parameter vector employed to simulate the model.

```
%model simulation for each sample of the Latin
   Hypercube
disp('Starting model simulation with perturbed
   parameters');
[AllResults,AllPerturbations]=
   start_simulation_NOSYMBIO(model,Nr,LBpi,UBpi,Ns);
disp('All done! Model simulation completed!');

Starting model simulation with perturbed parameters
    'Generated a Latin Hypercube of size 10000x6'
Starting parallel pool (parpool) using the 'local'
   profile ...
connected to 4 workers.
    'Completed one realization. The array of results
       has size 10000x1'
      ...

    'Generated a Latin Hypercube of size 10000x6'
    'Completed one realization. The array of results
       has size 10000x1'
Parallel pool using the 'local' profile is shutting
   down.
All done! Model simulation completed!
```

Then, this function computes the evaluation function with the method previously specified and then calculates the Moment Independent Robustness Indicator (MIRI) for the parameter vector.

```
%computation of the MIRI
disp('Starting computation of the MIRI for each
   parameter...');
try
  compute_MIRI_NOSYMBIO(model,variable_name,
      current_func,tail_size,current_tm,Nr,Ns,
      AllResults,AllPerturbations,folder)
catch ME
    break
end

Starting computation of the MIRI for each parameter...
saving results...
    'Array of MIRI has size 100x6'
```

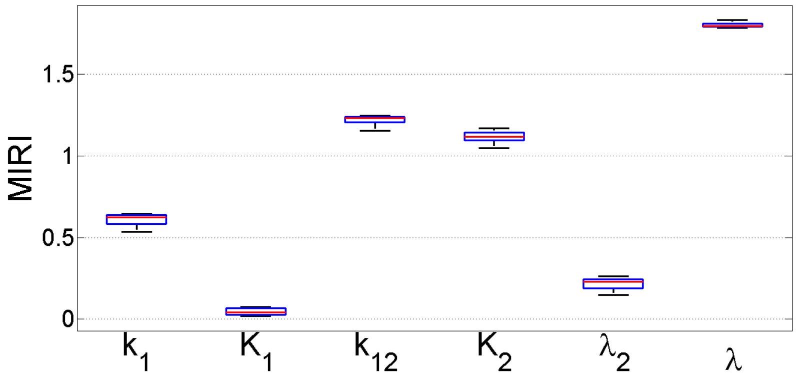

Figure 1.1: MIRIs generated by the CRA algorithm applied to the Pulse Generator model with *Area* as evaluation function. The same figure is shown in the main text (Figure 10).

```
saving mode of the conditional upper pdf of the parameter vector
    'The mode vector of the upper pdf has size 1x6'
saving mode of the conditional lower pdf of the parameter vector
    'The mode vector of the upper pdf has size 1x6'
saving the probability density function of the evaluation function
saving probability density functions of all parameters
Plot of the probability density function of the chosen evaluation function
```

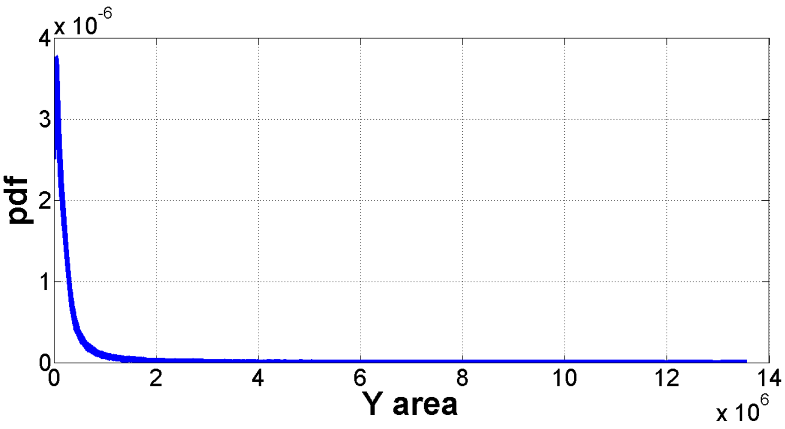

Figure 1.2: Pdf of the evaluation function generated by the CRA algorithm applied to the Pulse Generator model. The same figure is shown in the main text (Figure 10).

```
Plot of the parameter probability density functions
```

```
Elapsed time is ... seconds.
```

The next seven figures show the conditional probability density functions $f_{p_i|L}$ and $f_{p_i|U}$ for each parameter of the model. These conditional pdfs are employed for the calculus of the MIRIs in Figure 1.1.

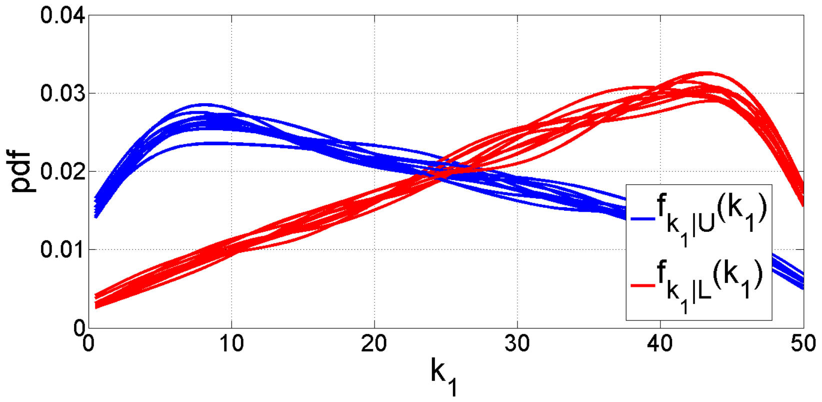

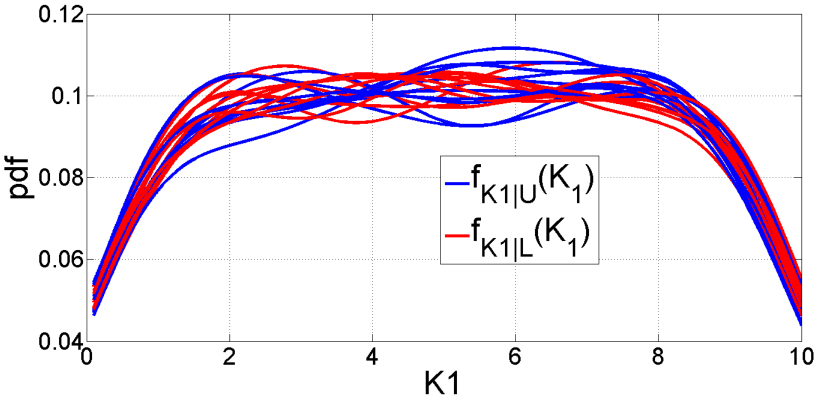

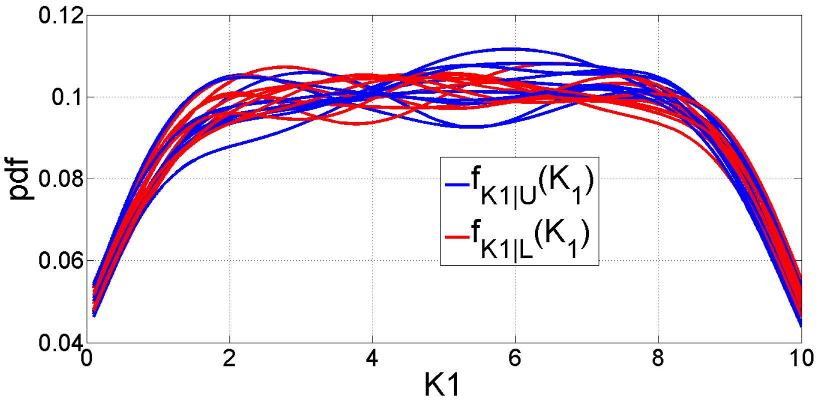

0.12
0.1
0.08
0.06
0.04
pdf
0
2
4
6
8
10
K1
$f_{K1|U}(K_1)$
$f_{K1|L}(K_1)$

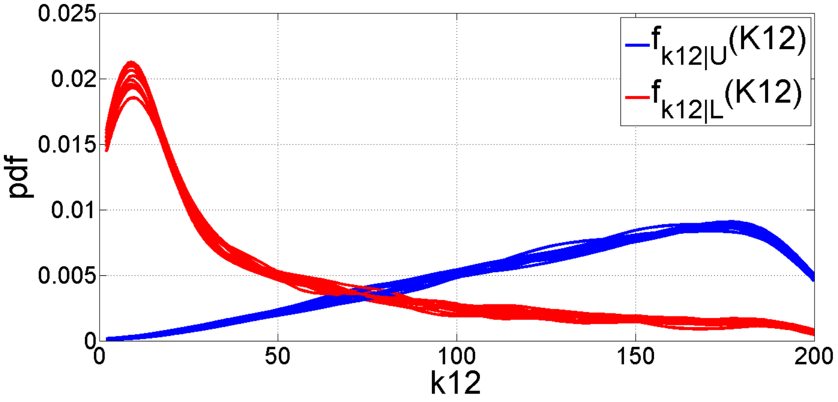

0.025
0.02
0.015
0.01
0.005
0
pdf
0
50
100
150
200
k12
$f_{k12|U}(K12)$
$f_{k12|L}(K12)$

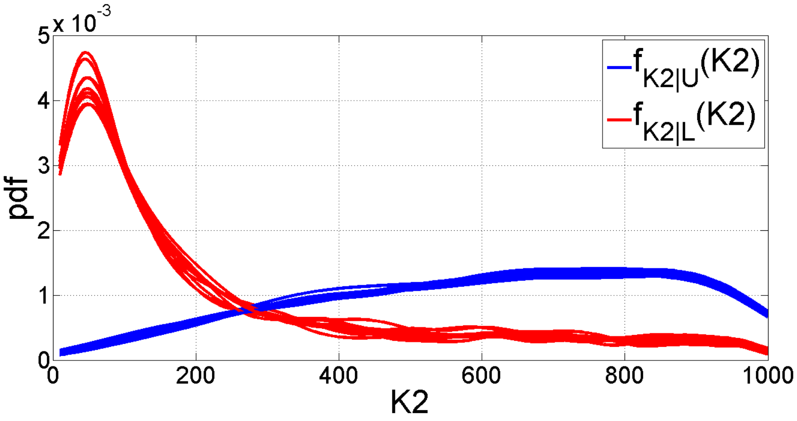

x 10^{-3}
5
4
3
2
1
0
pdf
0
200
400
600
800
1000
K2
$f_{K2|U}(K2)$
$f_{K2|L}(K2)$

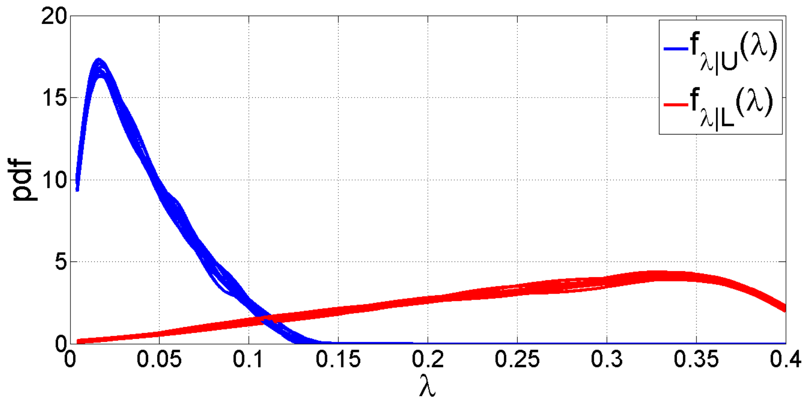

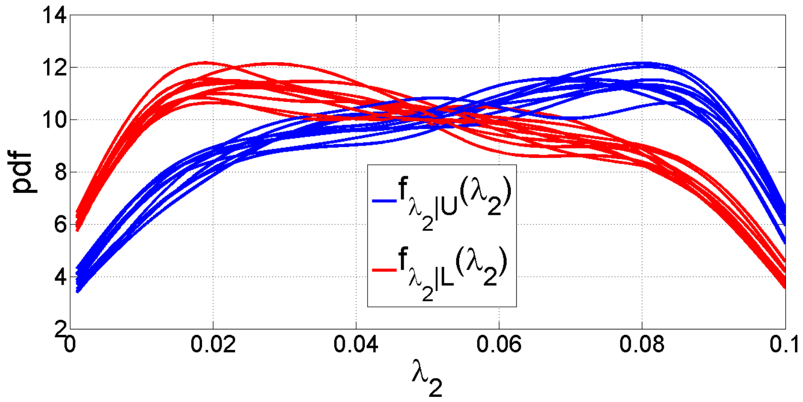

Tuning parameters of the CRA

In this section we explain the role of the tuning parameters of the CRA and how they should be properly set. As a rule of thumb, the upper and lower boundaries of the LHS should span the parameter space of at least two orders of magnitude because in this way the CRA algorithm is able to capture the behavior of the evaluation function when all the parameters are perturbed in a sufficiently wide range of values. As an example, we apply the CRA algorithm to the Pulse Generator Network, choosing as evaluation function the area under the curve of the output variable in two different scenarios:

1. the lower and upper boundaries of the LHS are set equal to 0.8 and 2, respectively and N_S is set to 10000;

2. the lower and upper boundaries of the LHS are set equal to 0.01 and 100, respectively and N_S is set to 100000.

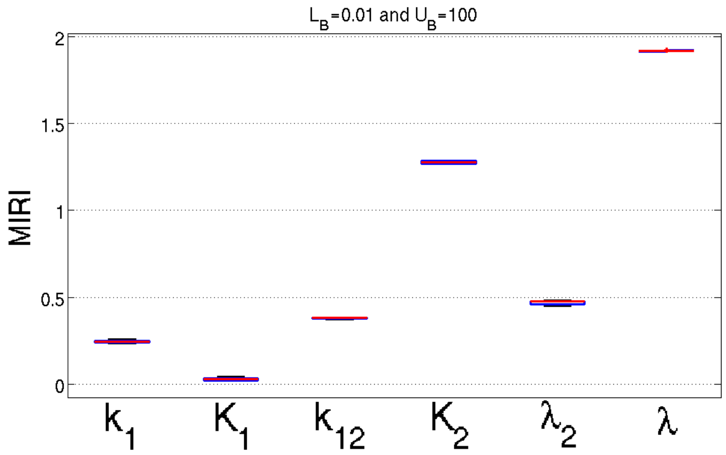

Figure 1.3: Results of the CRA algorithm when the lower and upper boundaries of the LHS are 0.01 and 100, respectively.

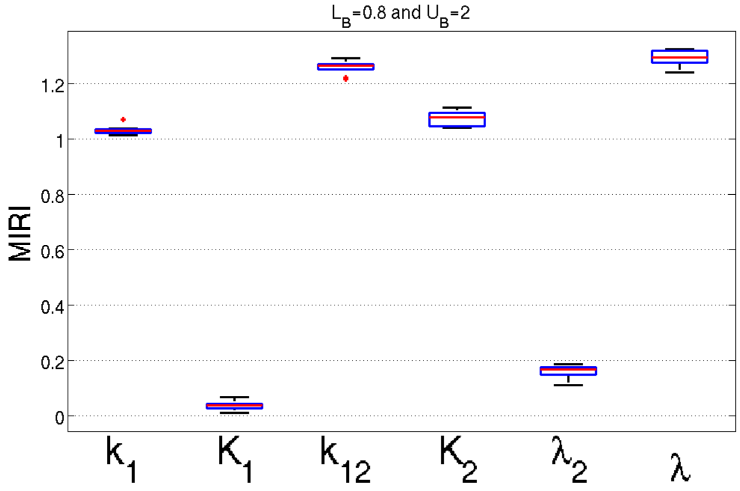

Figure 1.4: MIRI in output from CRA when the lower and upper boundaries of the LHS are 0.8 and 2, respectively.

From the Figure 1.3it is possible to see that the results of CRA are stable when the ranges of the LHS are wider, as long as N_S is sufficiently high to sample the parameter space in a comprehensive way. Parameter λ has the highest value of the MIRI as it was clear also from Figure 1.1.

On the other hand, if the lower and upper boundaries of LHS are too close to each other the results are not coherent. Indeed, from Figure 1.4 it is not possible to establish which is the parameter that most influences the behavior of the output variable because four parameters have similar MIRI values, i.e. k_1, k_{12}, K_2 and λ.
As regards the N_S parameter, it directly affects the estimation of the pdf of the evaluation function, $f_{Z_i}(z_i)$, and the conditional pdfs of the model parameters, $f_{p_i|L}$ and $f_{p_i|U}$. As it is stated in the paper of the CRA algorithm (see References), for a fixed level of probability α=0.1, N_S should be greater than or equal to 1000/α=10000. In order to show that, we apply again the CRA algorithm to the Pulse Generator Network, choosing as evaluation function the area under the curve of the output variable. We fix the values of the lower and upper boundaries of the LHS equal to 0.1 and 10 respectively, as in the main text of the paper. Then we vary N_S by setting it once equal to 3000 and once to 100000.

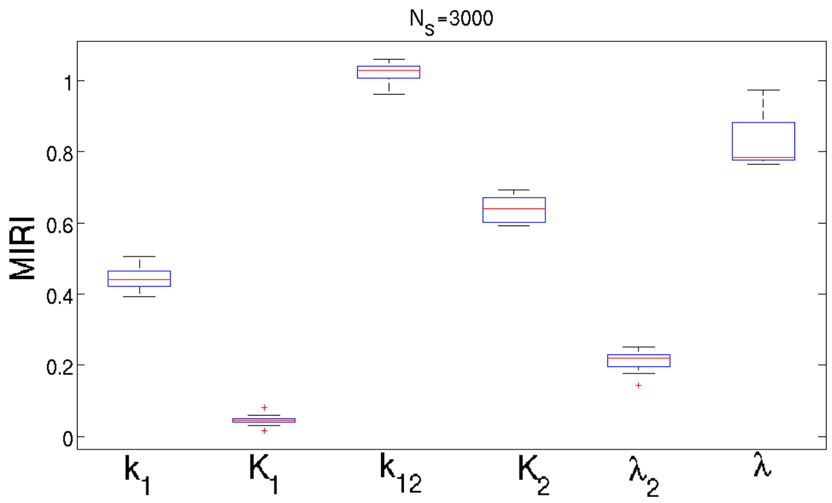

Figure 1.5: MIRIs in output from the CRA when parameter N_S is equal to 3000 and the lower and upper boundaries of the LHS are 0.1 and 10 respectively.

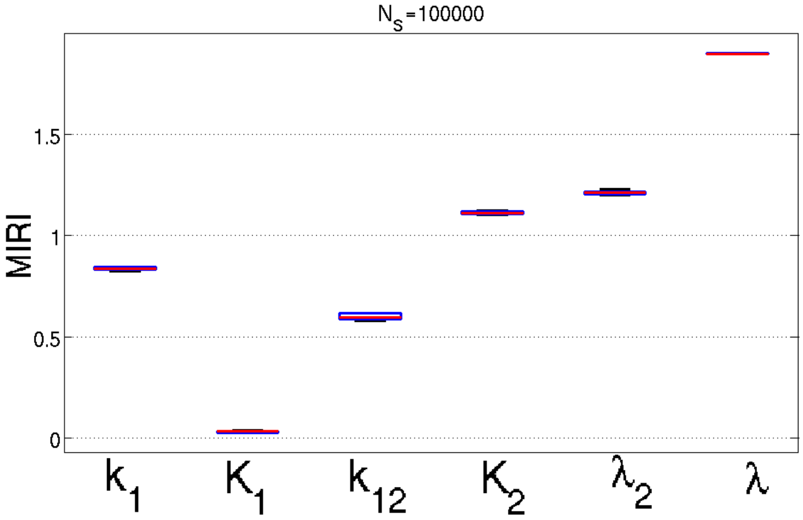

Figure 1.6: MIRIs in output from the CRA when parameter N_S is equal to 100000 and the lower and upper boundaries of the LHS are 0.1 and 10 respectively.

In the first case the value of N_S is too small and actually the CRA algorithm is computing just a subsample of all the parameter space. Comparing MIRIs in Figure 1.1 with those in Figure 1.5, we can see that the parameter with the highest value of the MIRI is not the same. In more details, when N_S is equal to 10000, parameter λ has the strongest impact on the behavior of the output variable Y. When N_S is equal to 3000 this is not true because the MIRI value of parameter λ is strongly reduced. As a consequence the results are not coherent. On the other hand, when N_S is equal to 100000, we can see from Figure 1.1 and Figure 1.6 that the MIRIs are almost identical. This proves that the algorithm is robust against the variation of N_S, provided that it is not below the suggested value.
Moreover, as it is intuitive, the greater is N_S and the greater is the time required by the CRA algorithm to terminate because N_S corresponds to the number of times that the mathematical model has to be integrated. Table 1.1 summarizes the time required to complete one realization of the CRA algorithm under the different scenarios shown above.

Table 1.1: Computational cost to run the CRA algorithm for the different settings of the tuning parameters.

L_B	U_B	N_S	Time (sec.)
0.1	10	3000	10
0.1	10	100000	1500
0.8	2	10000	25
0.01	100	10000	1000

References

Bianconi, F., Baldelli, E., Luovini, V., Petricoin, E. F., Crin, L., and Valigi, P. (2015). *Conditional robustness analysis for fragility discovery and target identification in biochemical networks and in cancer systems biology.* BMC systems biology, 9(1), 70.
